# The Complex Hodological Architecture of the Macaque Dorsal Intraparietal Areas as Emerging from Neural Tracers and DW-MRI Tractography

**DOI:** 10.1523/ENEURO.0102-21.2021

**Published:** 2021-07-02

**Authors:** Roberto Caminiti, Gabriel Girard, Alexandra Battaglia-Mayer, Elena Borra, Andrea Schito, Giorgio M. Innocenti, Giuseppe Luppino

**Affiliations:** 1 Neuroscience and Behavior Laboratory, Istituto Italiano di Tecnologia, Rome 00161, Italy; 2 CIBM Center for Biomedical Imaging, Lausanne CH-1015, Switzerland; 3 Radiology Department, Centre Hospitalier Universitaire Vaudois and University of Lausanne, Lausanne CH-1011, Switzerland; 4 Signal Processing Laboratory (LTS5), École Polytechnique Fédérale de Lausanne, Lausanne, CH-1015 Switzerland; 5 Department of Physiology and Pharmacology, University of Rome SAPIENZA, Rome 00185, Italy; 6 Dipartimento di Medicina e Chirurgia, Università di Parma, Parma 43125, Italy; 7 PhD Program in Behavioral Neuroscience, University of Rome SAPIENZA, Rome 00185, Italy; 8 Department of Neuroscience, Karolinska Institutet, Stockholm 171 1777, Sweden; 9 Brain and Mind Institute, École Polytechnique Fédérale de Lausanne, Lausanne CH-1015, Switzerland

**Keywords:** cortico-cortical connections, diffusion tractography, frontal cortex, intraparietal sulcus, macaque brain, parietal cortex

## Abstract

In macaque monkeys, dorsal intraparietal areas are involved in several daily visuomotor actions. However, their border and sources of cortical afferents remain loosely defined. Combining retrograde histologic tracing and MRI diffusion-based tractography, we found a complex hodology of the dorsal bank of the intraparietal sulcus (db-IPS), which can be subdivided into a rostral intraparietal area PEip, projecting to the spinal cord, and a caudal medial intraparietal area MIP lacking such projections. Both include an anterior and a posterior sector, emerging from their ipsilateral, gradient-like connectivity profiles. As tractography estimations, we used the cross-sectional area of the white matter bundles connecting each area with other parietal and frontal regions, after selecting regions of interest (ROIs) corresponding to the injection sites of neural tracers. For most connections, we found a significant correlation between the proportions of cells projecting to all sectors of PEip and MIP along the continuum of the db-IPS and tractography. The latter also revealed “false positive” but plausible connections awaiting histologic validation.

## Visual Abstract

**Figure:**
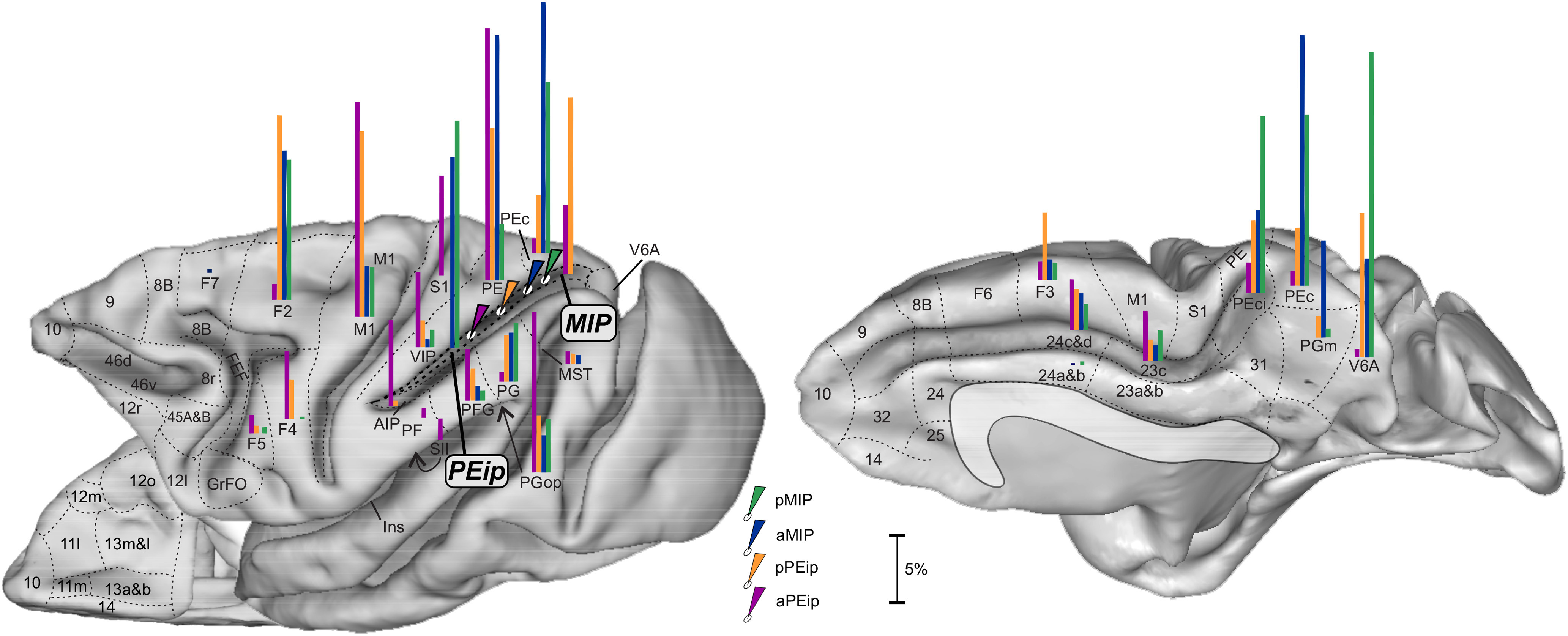

## Significance Statement

Combined histologic and diffusion-weighted MRI (DW-MRI) tractography revealed that areas PE intraparietal (PEip) and Medial intraparietal (MIP) share common inputs from other parietal, frontal and, to a lesser extent, cingulate areas, although with different gradient-like connectivity profiles. Both tractography and histology revealed a high number of common paths, although tractography showed false positive connections awaiting histologic validation. A correlation was performed between the proportion of labeled cells projecting to PEip and MIP and the diffusion-based connectivity estimation of the regions of interest (ROIs) corresponding to the injection sites of retrograde tracers. The results showed a significant correlation from most connections studied, opening a window for future studies contrasting proportions of cells, giving rise to the fiber bundles connecting cortical areas, with measures of diffusion tractography connectivity.

## Introduction

Areas PE intraparietal (PEip) and medial intraparietal (MIP) in the dorsal bank of the intraparietal sulcus (db-IPS) of monkeys are two crucial nodes for controlling visuomotor behavior. This view stems from different sources of information. The first relates to their input-output relationships (Johnson et al., 1996; Caminiti et al., 1996; Matelli et al., 1998; Marconi et al., 2001; Bakola et al., 2017; Battaglia-Mayer and Caminiti, 2018, 2019), since they receive projections from visuomotor areas V6A and PGm and project to premotor and motor cortex (see Caminiti et al., 2017). The second consists in the functional properties of their neurons (see Lacquaniti et al., 1995; Johnson et al., 1996; Batista et al., 1999), which combine retinal signals about target location, with eye and hand position and movement directions within their tuning fields (Battaglia-Mayer et al., 2000, 2001). The third stems from the consequences of lesions of parieto-occipital areas in humans, consisting in a defective visual control of reaching, known as optic ataxia (Bálint, 1909; see Rossetti and Pisella, 2018).

To date, aspects of PEip and MIP connectivity remain unknown, since the difficulty of injecting histologic tracers over the entire dorsoventral (D-V) extent of the IPS rendered only a partial view of its connectivity. Previous attempts to mark the PEip/MIP border were based on the presence of cortico-spinal projections (Matelli et al., 1998) or on myeloarchitectonic criteria (Bakola et al., 2017). Based on cytoarchitectonics, Pandya and Seltzer (1982) labeled this region of the superior parietal lobule (SPL) as area PEa, to distinguish it from the remaining part of area 5. This study was, however, antecedent to the identification of MIP as the dorsal intraparietal region projecting to area PO (Colby et al., 1988).

The difficulties of histologic studies can tentatively be overcome by diffusion-weighted MRI (DW-MRI) tractography. Albeit known limitations, such as the identification of false-positive connections and biases toward reconstructing short and strong connections (Jones et al., 2013; Van Essen et al., 2014; Jbabdi et al., 2015; Knösche et al., 2015; Maier-Hein et al., 2017; Aydogan et al., 2018; Jeurissen et al., 2019; Schilling et al., 2019a,b; Girard et al., 2020), tractography shows promising results when compared with histology (Dauguet et al., 2007; Dyrby et al., 2007; Seehaus et al., 2013; Jbabdi et al., 2015; Thomas et al., 2014; Azadbakht et al., 2015; Calabrese et al., 2015; Gyengesi et al., 2015; van den Heuvel et al., 2015; Knösche et al., 2015; Donahue et al., 2016; Delettre et al., 2019; Ambrosen et al., 2020; Girard et al., 2020). Particularly, Calabrese et al. (2015), Donahue et al. (2016), and Ambrosen et al. (2020) have reported positive results when comparing labeled cells count from tracer injections in the monkey brain with connectivity weights derived from DW-MRI tractography.

In this study, we combined tractography and histology to elucidate the connectivity of PEip and MIP. In two macaque monkeys, we injected different retrograde fluorescent tracers along the antero-posterior (A-P) extent of the db-IPS and established their putative border based on the distribution of cortico-spinal cells projecting to the cervical segments of the spinal cord, as determined in two other animals (see Matelli et al., 1998). The connectivity of the db-IPS was studied with tractography in a fifth animal and compared in a quantitative fashion with histologic data. To explore potential connections of PEip and MIP not yet revealed by tract tracing studies, the D-V extent of these areas was subdivided into different regions of interest (ROIs). This was inspired by earlier anatomo-functional studies (Johnson et al., 1996; Battaglia-Mayer et al., 2001) showing systematic changes of both functional properties and corticocortical connectivity in the D-V extent of the intraparietal cortex.

Combining histology and tractography revealed a significant correlation between the proportion of cells projecting to MIP and/or PEip and the diffusion-based connectivity estimates of the corresponding streamlines. Furthermore, tractography resulted to be very useful in revealing aspects of parietal connectivity which could not be explored based on neural tracer injections. Beyond advancing the information about cortical connectivity of the IPS, these results offer a quantitative cross-validation of the two methods and call for a histologic validation of predictions emerging from tractography.

## Materials and Methods

### Neural tracer experiments

#### Subjects

The tracer experiments were conducted in four male monkeys. In two animals (*Macaca mulatta*; cases 72 and 73; body weight 12 and 12.50 kg, respectively) retrograde neural tracers were injected at different A-P levels of the db-IPS. Additional data from two *Macaca nemestrina* (cases 10 and 21; body weight 5.2 and 4.4 kg, respectively), in which a retrograde tracer was injected in the lateral funiculus of the spinal cord, were used for visualizing the origin of corticospinal projections from the db-IPS. Data from these two cases have been already partially used in previous studies (Luppino et al., 1994; Matelli et al., 1998; Rozzi et al., 2006; Borra et al., 2010).

Animal handling as well as surgical and experimental procedures complied with the European law on the humane care and use of laboratory animals (Directives 86/609/EEC, 2003/65/CE, and 2010/63/EU) and the Italian laws in force regarding the care and use of laboratory animals (D.L. 116/92 and 26/2014). All procedures were approved by the Veterinarian Animal Care and Use Committee of the University of Rome SAPIENZA or of the University of Parma, and then authorized by the Italian Ministry of Health.

#### Surgical procedures

Surgery was performed under aseptic conditions. Cases 72 and 73 were preanaesthetized with ketamine (5 mg/kg, i.m.) and dexmedetomidine hydrochloride (0.1 mg/kg, i.m.), intubated and anaesthetized with a mix of oxygen/isoflurane (1–3% to effect). Lidocaine (2%) was used locally to minimize pain during skin incision in the scalp. Desametasone (6 mg/kg) was given before dura opening, to prevent brain inflammation and edema. The skull was then trephined over the target region, and the dura was opened to expose the intraparietal sulcus. A constant infusion of Fentanil (0.2 mg/kg/h, i.v.) was performed until the end of the surgical procedures. The selection of the injection sites was based on identified anatomic landmarks, such as the rostral tip of the IPS. In cases 10 and 21, in which tracers were injected in the spinal cord, under general anesthesia (5 mg/kg ketamine, i.m., and 0.08–0.1 mg/kg medetomidine, i.m.), following a laminectomy, the dura was opened, and the segment of the spinal cord selected for the injection exposed. During all surgeries, hydration was maintained with saline, and temperature using a heating pad. Heart rate, blood pressure, respiratory depth, O_2_ saturation, and body temperature were continuously monitored.

#### Tracer injections

**Figure 1.**
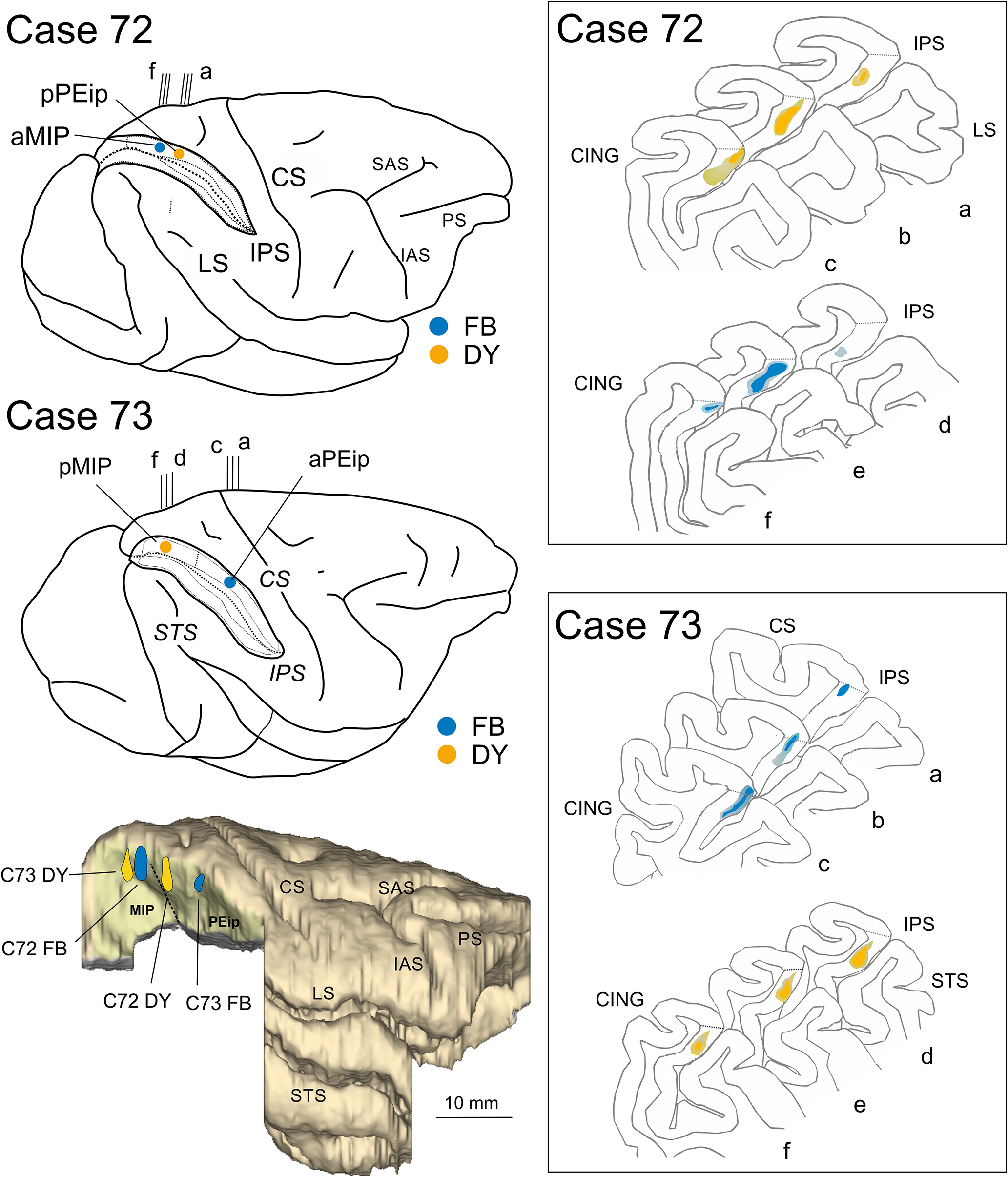
Brain figurines in the top and middle left part of the figure and the corresponding histologic sections on the right show the location of the FB and DY injection sites along the db-IPS (IPS) in cases 72 and 73. Case 73 is shown as a right hemisphere. The IPS is shown as “opened” to better visualize the dorsal and ventral banks. pPEip and aPEip indicate anterior and posterior part of area PEip, respectively. The same applies to area MIP (aMIP, pMIP). In the section drawings, the injection sites are shown as a deep colored zone corresponding to the core surrounded by a light-colored zone corresponding to the halo. The bottom left part of the figure shows a 3D reconstruction of a right hemisphere in which the inferior parietal lobule (IPL), including the ventral bank of the IPS was removed to show in a single comprehensive image the relative A-P locations of the four tracer injections (blue and yellow spots) in the different parts of areas MIP and PEip. CS, STS, LS, PS, SAS/IAS, and CING indicate central, superior temporal, lateral, principal, superior/inferior arcuate, and cingulate sulci.

Once the appropriate site was chosen, fluorescent tracers [fast blue (FB) 3% in distilled water, diamidino yellow (DY) 2% in 0.2 m phosphate buffer at pH 7.2, both from Illing Plastics GmbH] were slowly pressure injected with a glass micropipette attached to the needle of a Hamilton microsyringe at different depths and A-P levels in the medial bank of the IPS. In case 72 (Fig. 1), FB (two deposits, 0.15 μl each, at a depth of 3 and 4 mm, in the anterior part of area MIP, aMIP) and DY (two deposits, 0.15 μl each, at a depth of 3 and 4 mm, in the posterior part of area PEip, pPEip) were injected at ∼16 and 13 mm caudal to the rostral end of the right IPS, respectively. In case 73 (Fig. 1), FB (0.3 μl) and DY (0.3 μl) were injected at a depth of 4 mm, caudal to the rostral end of the left IPS, at ∼8.5 mm (in the anterior part of area PEip, aPEip) and 18 mm (in the posterior part of area MIP, pMIP), respectively. To facilitate comparison of the data with case 72, the brain in case 73 is shown as a right hemisphere. After the tracer injections were placed, the dura flap was sutured, the bone was replaced, and the superficial tissues were sutured in layers.

**Figure 2.**
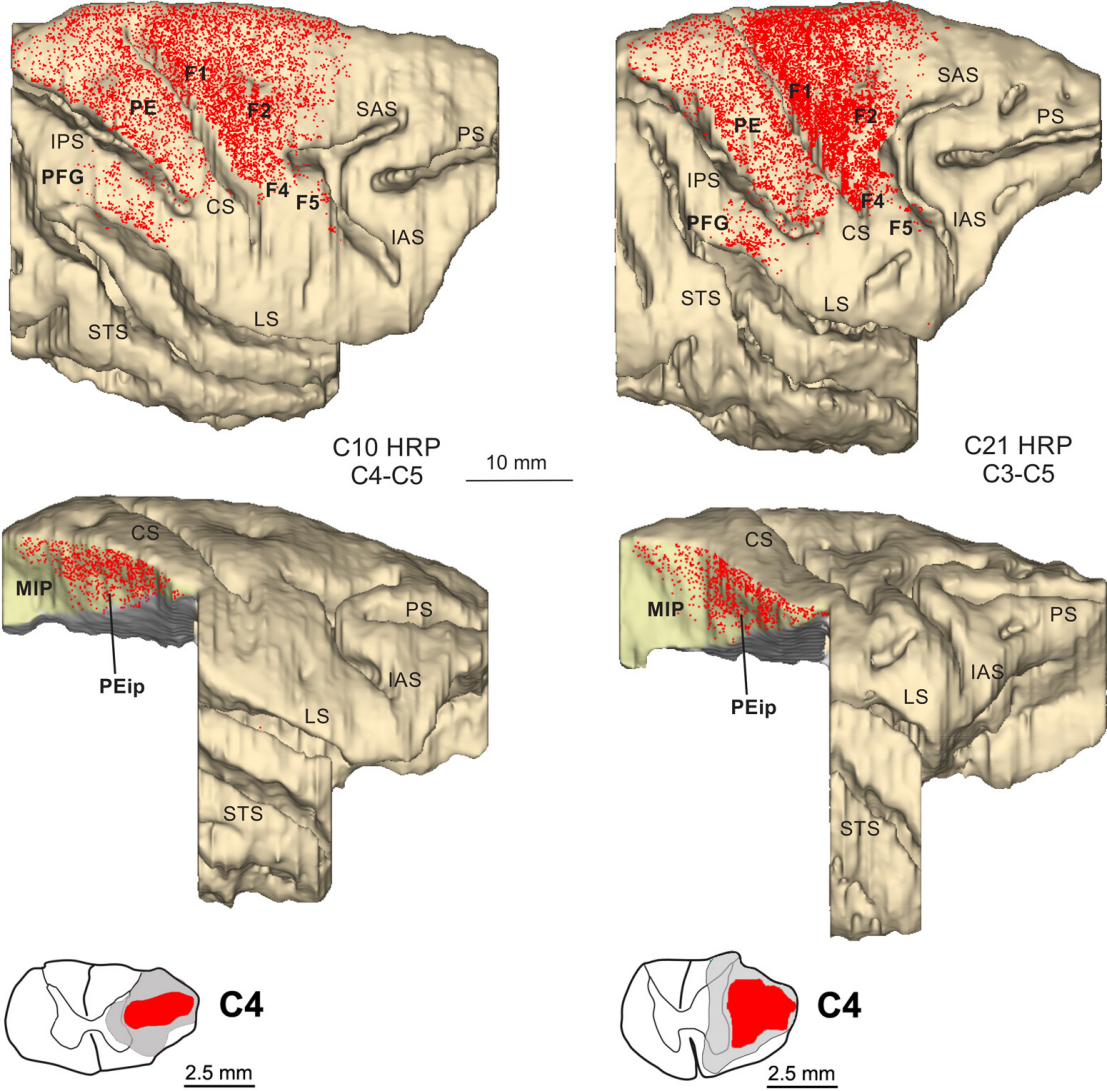
Distribution of retrogradely-labeled cells (RLCs) observed following HRP injections in the lateral funiculus of the spinal cord at upper cervical levels in cases 10 and 21, shown in dorsolateral views of the 3D reconstructions of the injected hemispheres and lateral views of the db-IPS exposed after dissections of the IPL and of part of temporal lobe. Each dot corresponds to one labeled neuron. In the lower part of the figure, coronal sections through C4 level of the spinal cord show the HRP injection core (in red) and halo (in gray). Other abbreviations as in Figure 1.

In cases 10 and 21 the retrograde tracer horseradish peroxidase (HRP; 30% in 2% lysolecithin, Sigma-Aldrich) was pressure injected with a 5-μl Hamilton microsyringe in the left lateral funiculus in both monkeys (Fig. 2). In one animal (case 10), the tracer (multiple injections, total amount 10 μl) was injected at the C4–C5 spinal level, in the other (case 21, multiple injections, total amount 15 μl) at C3–C5 level. Upon the completion of the injections, the spinal cord was covered with Gelfoam and wounds were closed in layers.

Upon recovery from anesthesia, the animals were returned to their home cages and closely monitored. Dexamethasone and prophylactic broad-spectrum antibiotics were administered preoperatively and postoperatively. Furthermore, analgesics were administered intraoperatively and postoperatively.

### Histologic procedures

At the end of the survival time (26 d for case 72; 23 d for case 73; 3 d for cases 10 and 21), the animals were given a dose of atropine (0.4 ml, i.m.) and diazepam (2 ml valium, i.m.), preanaesthetized as above, and received an intravenous lethal injection of sodium thiopental (200 mg/kg, i.v). They were perfused through the left cardiac ventricle with saline, 4% paraformaldehyde, and 5% glycerol in this order. All solutions were prepared in phosphate buffer 0.1 m, pH 7.4. Each brain was then blocked coronally on a stereotaxic apparatus, removed from the skull, photographed, and placed in 10% buffered glycerol for 3 d and 20% buffered glycerol for 4 d. Finally, each brain was cut frozen in coronal sections 60 μm thick. In cases 10 and 21, the spinal cord was cut in 60-μm-thick coronal sections. In cases 72 and 73, one series of every fifth section was mounted, air-dried, and quickly cover-slipped for fluorescence microscopy. In cases 10 and 21, one series of every fifth section through the right hemisphere and the brainstem, and every tenth section through the spinal cord was processed for HRP histochemistry using tetramethylbenzidine as chromogen (Mesulam, 1982). Sections were rinsed in 0.01 m acetate buffer, pH 3.3, and developed at 4°C in a solution of 0.09% sodium nitroferricyanide, 0.005% tetramethylbenzidine, and 0.006% hydrogen peroxide in 0.01 m acetate buffer. Finally, one series of every fifth section in all brains and of every tenth section in the spinal cord in cases 10 and 21, was stained with the Nissl method (0.1% thionin in 0.1 m acetate buffer, pH 3.7).

### Injection sites and distribution of retrogradely labeled neurons

In cases 72 and 73, the FB and DY injection sites, defined according to Kuypers and Huisman (1984) and Condé (1987), were completely restricted to the cortical gray matter, involving almost the entire cortical thickness, or at least layers III–V. Injection sites were then attributed to area PEip or MIP, as defined from the distribution of corticospinal labeled neurons in the db-IPS (cases 10 and 21), as detailed in Table 1.

**Table 1.** Percentages (%) and total number (*n*) of labeled neurons observed after tracer injections in MIP and PEip

Injected area	aPEip	pPEip	aMIP	pMIP
Case	73FB	72DY	72FB	73DY
*Prefrontal*				
12r, 12l, 12m&12o, 11m&11l, 13,GrFO, 10, 31, 32, 24, 25, 14, 9, 45A&B, 46d, 46v, 8B, 8r&FEF	–	–	–	–
*Frontal*				
F6	–	0.2	–	–
F7	–	0.3	0.1	–
F3	1.4	4.9	1.6	1.3
F2	1.2	13.4	10.9	10.2
F5	1.5	0.7	0.2	0.5
F4	5.0	2.9	–	0.2
M1 (F1)	15.6	13.5	3.8	3.7
*Cingulate*				
24c&d	3.7	3.0	2.7	2.0
24a&b	–	0.2	0.1	0.3
23a&b	–	–	–	–
23c	3.7	1.6	1.2	2.3
*Somatosensory*				
SI	7.3	–	–	–
SII	1.6	0.2	–	–
Insular	1.7	–	–	–
*Superior parietal lobule (SPL)*				
PE	18.3	11.1	17.8	4.1
PEc	1.1	4.2	18.2	12.5
PEci	2.2	5.3	6.1	12.9
PGm	–	1.6	7.1	0.7
V6A	0.7	10.5	7.3	22.2
*Intraparietal (IPS)*				
PEip	**X**	**X**	13.8	16.5
MIP	5.1	12.9	**X**	**X**
AIP	6.3	0.5	–	0.2
VIP	5.5	2	0.7	1.3
LIP	–	–	–	–
*Inferior parietal lobule (IPL)*				
PF	0.7	–	–	–
PFG	3.8	2.4	1.1	0.8
PG	0.7	3.4	3.6	4.3
Opt	–	–	–	–
PGop	11.7	4.2	2.8	3.9
*Temporal*				
MST	1	0.8	0.7	–
MT	–	–	–	–
Tpt	0.2	–	0.1	–
**Total number of labelled cells (n)**	**20,556**	**62,312**	**21,927**	**61,135**

Injection sites are sorted relative to their A-P position along the db-IPS, to better display the gradient-like distribution of their projections (–, labeling < 0.1% or no labeling). No cell counts are reported for the areas containing the injection sites (X).

The cortical distribution of FB-retrogradely and DY-retrogradely labeled cells (cases 72 and 73), as well as of HRP-labeled cells (cases 10 and 21), here referred to as retrogradely-labeled cells (RLCs), was plotted in sections every 600 μm (300 μm in cases 10 and 21). In each examined section the outer and inner cortical borders and the location of each labeled neuron were plotted with the aid of inductive displacement transducers mounted on the *x*- and *y*-axes of the microscope stage. The transducer signals were digitized and stored by using software developed in our laboratory that allows the visualization of section outlines, of gray-white matter borders, and of labeled cells.

Data from individual sections were then imported into the three-dimensional (3D) reconstruction software developed in house (Demelio et al., 2001) to create volumetric reconstructions of the hemispheres from individual histologic sections containing connectional and/or architectonic data and providing realistic visualizations of the labeling distribution. The distribution of RLCs on exposed cortical surfaces was visualized in mesial and dorsolateral views of the hemispheres, whereas that in the db-IPS in lateral views of the hemispheres, in which the bank was exposed with dissection of the inferior parietal lobule (IPL) and the temporal lobe.

The nomenclature and the map adopted for the areal attribution of the labeled neurons was the same of that used in a recent quantitative study of the connectivity of the parieto-frontal system (Caminiti et al., 2017). Briefly, the superior and medial parietal cortex was defined according to architectonic criteria described in Pandya and Seltzer (1982) and Luppino et al. (2005), while area MIP was defined based on the distribution of corticospinal projections (see Results). In the IPL the gyral convexity areas were defined according to cytoarchitectonic and chemoarchitectonic criteria described in Gregoriou et al. (2006) and those of the lateral bank of the intraparietal sulcus based on connectional criteria described in Borra et al. (2008). The fundal region of the intraparietal sulcus was assigned to the ventral intraparietal (VIP) area as defined by Colby and Duhamel (1991). In the frontal lobe, frontal and cingulate motor areas were defined according to architectonic criteria described in Matelli et al. (1985, 1991) and Belmalih et al. (2009). Prefrontal areas were defined according to Carmichael and Price (1994), Gerbella et al. (2007), and Saleem et al. (2014).

### Quantitative analysis and laminar distribution of retrograde labeling

In all the cases, we counted the number of RLCs plotted in the ipsilateral hemisphere, beyond the limits of the injected area, in sections at every 600-μm interval. Cortical afferents to areas PEip or MIP were then expressed in terms of the percentage of labeled neurons found in a given cortical subdivision, with respect to the overall retrograde labeling found for each tracer injection. As for the brain parcellation adopted in this study, for both histologic and tractography data, see dedicated paragraph below.

Furthermore, to obtain information about the laminar patterns of the observed connections, the labeling attributed to a given area and reliably observed across different sections and cases was analyzed in sections at every 300 μm in terms of amount of RLCs located in the superficial (II–III) versus deep (V–VI) layers.

### DW-MRI experiment

#### Brain processing for *ex vivo* DW-MRI acquisition

The DW-MRI data from *ex vivo* brain of a male *Macaca mulatta* (M105, four years and 10 months old, 10.1 kg body weight) available from Ambrosen et al. (2020) was used. The brain was perfused following the protocol illustrated in Ahmed et al. (2012) and prepared for MRI *ex vivo* scanning as described by Dyrby et al. (2011). The DW-MRI data were acquired at 0.5 mm isotropic resolution. The data were sampled in 180 uniformly distributed directions on each of three b-value shells (b = [1.477, 4.102, 8.040] ms/μm^2^) and nine non-diffusion-weighted images (b = 0 ms/μm^2^). The protocol was repeated twice and averaged before further processing (for more details on the MRI acquisition protocol, see Ambrosen et al., 2020). We also used the midcortical surface from Ambrosen et al. (2020). The fiber orientation distributions were estimated using the multi-shell multi-tissue constrained spherical deconvolution algorithm available in the MRtrix3 software (Jeurissen et al., 2014; Tournier et al., 2019). The brain partial volume estimates for the white matter, gray matter, and cerebrospinal fluid were obtained from the averaged non-diffusion-weighted image using the FSL Fast software (Zhang et al., 2001).

#### Brain parcellation

We used the brain parcellation of the right hemisphere available in Girard et al. (2020). Fifty-nine cortical areas were manually parcellated following the study by Caminiti et al. (2017), on the animal used for the *ex vivo* DW-MRI acquisition. Areas 46dr and 46dc were grouped in a single ROI, 46d. Similarly, we grouped areas 46vr, r46vc, c46vc in ROI 46v, areas c12r, i12r, r12r in ROI 12r, areas 9l, 9m in ROI 9, areas 45A, 45B in ROI 45, areas 8Ad, 8Av in ROI 8r&FEF, areas F7-PMdr, F7-SEF in ROI F7, areas F2vr, F2preCD in ROI F2, areas F5p, F5a/44, F5c in ROI F5. Areas 24 and 25, the insula and Tpt were added to cortical parcellation based on atlases of the rhesus monkey brain (Paxinos et al., 2009; Saleem and Logothetis, 2012). Together, these cortical areas make 48 ROIs for investigating the connectivity of PEip and MIP. To obtain a detailed parcellation of the db-IPS, we first merged area PEip and MIP in a single area. This resulted in 38 A-P MRI coronal slices (from #105 to #68; each 0.5-mm thickness) of the bank, which was then divided into three equally wide sectors: dorsal, middle, and ventral. However, the most anterior part of the area PEip was excluded from the fine parcellation of the db-IPS, because of the difficulty in identifying three sectors. The parcellation was done in the native MRI image space. The MRI images were manually aligned to the stereotaxic plane of the histologic sections for visual inspection.

#### DW-MRI tractography and connectivity

Probabilistic streamline tractography was performed using the Particle Filtering Tractography algorithms (Girard et al., 2014) available in the DIPY software library (Garyfallidis et al., 2014). Tractography was initiated in all white matter voxels using 25 seeds per voxel (9,713,750 seeds). Streamlines with a length superior to 2 mm in the white matter volume were used as input to the Convex Optimization Modelling for Microstructure Informed Tractography (COMMIT) method (Daducci et al., 2015). COMMIT was used to estimate each streamline contribution (weights) to the intra-axonal MRI signal fraction following the Stick–Zeppelin–Ball white matter microstructure model (Panagiotaki et al., 2012; Daducci et al., 2015). The tractography and microstructure estimation was repeated four times, resulting in a total of 23,137,312 streamlines and weights. All streamlines with an endpoint located in one of the 48 cortical ROIs and an endpoint in the A-P coronal slices of the db-IPS were selected for the diffusion-based connectivity analysis. Streamlines were selected using the MRtrix3 *tck2connectome* (Tournier et al., 2019) command, identifying connected ROIs with a radial search of 1 mm around streamlines endpoints. This resulted in 73,390 streamlines connecting the db-IPS to the cortical areas (dorsal: 29,378; middle: 24,474; ventral: 19,538).

#### Diffusion-based connectivity estimation

To cover a similar extent as the tracer injections, we merged the dorsal and middle sectors of our 3-fold subdivision of the db-IPS. We used a sliding window of five MRI coronal slice (2.5 mm) moving in the A-P direction selecting all streamlines connecting the merged sectors of the window to the cortical ROIs. From the 38 coronal MRI slices (#105 to #68), we obtained 34 windows in the A-P extent of the db-IPS, with each window made of five consecutive MRI slices (centered at slices #103 to #70, the two bordering slices at each extremity of the db-IPS were excluded). For each sliding window and each cortical ROI, we computed the sum of the COMMIT weights (i.e., estimation of the intra-axonal MRI signal fraction) associated with streamlines interconnecting them.

The diffusion-based connectivity distribution of a sliding window (dorsal and middle sectors of the db-IPS of five consecutive coronal MRI slices) was obtained by dividing each ROI’s weight by the sum of the weights associated with streamlines connecting that window to all cortical ROIs. The Pearson’s correlation coefficient was used to compare the diffusion-based connectivity distribution of each window with the histologic cell count distributions of the four injection sites.

## Results

### Neural tracers study

#### Subdivision of the db-IPS and location of the injection sites

The location of the injection sites placed at different A-P levels in the db-IPS and involving the bank for several mm in depth (cases 72 and 73) is shown in Figure 1. To assign injection sites and RLCs in the db-IPS to specific cortical entities, as in Matelli et al. (1998), we subdivided this region based on the distribution of corticospinal neurons, which clearly distinguishes between a rostral and a caudal sector (Fig. 2).

The upper part of Figure 2 shows the overall distribution on the dorsolateral cortical surface of the corticospinal labeled neurons observed after the injection of HRP in the lateral funiculus at the upper cervical levels (cases 10 and 21). The extensive labeling observed in both cases all over the precentral and postcentral gyri, except their most lateral part, suggested complete, or almost complete involvement of the contralateral lateral funiculus by the HRP injection. In the lower part of Figure 2, lateral views of the two hemispheres show the distribution of the RLCs observed in the db-IPS. In both hemispheres, the rostral part of the bank hosted the highest number of them, as compared with its caudal part, from the crown to the fundus. This rostral sector, which does not appear to project to the thoraco-lumbar spinal cord (Matelli et al., 1998) and hosts neurons dysinaptically connected with hand motorneurons (Rathelot et al., 2017), has been here referred to as PEip, according to the original definition of Matelli et al. (1998). Caudal to PEip, corticospinal neurons appeared to be confined to the uppermost part of the bank, which, therefore, for most of its extent lacked these projections. This last sector as a whole has been here referred to as area MIP. The border between PEip and MIP tended to run obliquely, from a ventrorostral to a dorsocaudal position and, at about half of the depth of the bank appeared to be located at an A-P level of ∼13 mm caudal to the rostral tip of the IPS. In the caudalmost part of the bank, MIP borders caudally with V6A (Luppino et al., 2005; Bakola et al., 2017).

#### Ipsilateral cortical projections to area MIP

**Figure 3.**
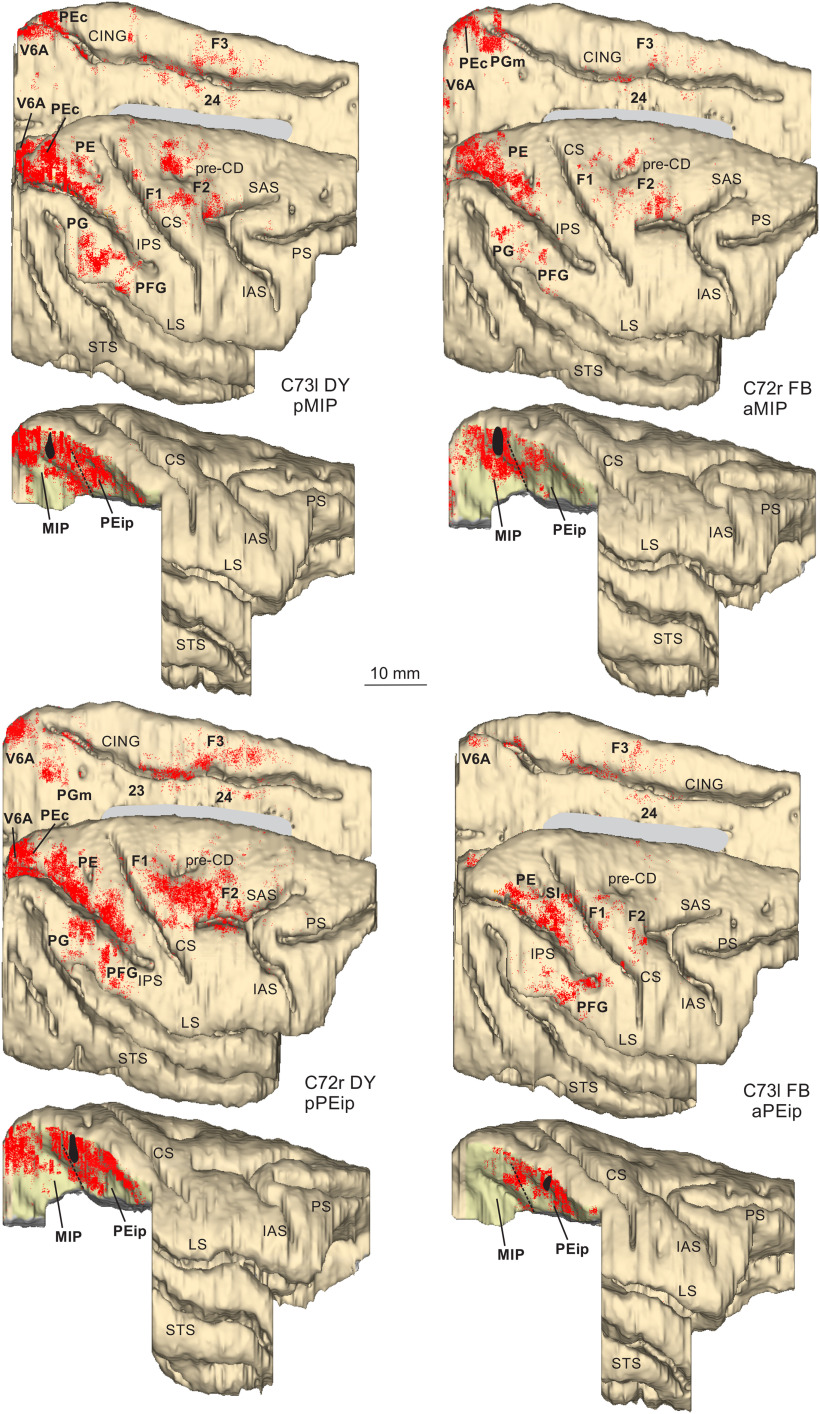
Distribution of RLCs observed following tracer injections in the db-IPS, shown in dorsolateral and mesial views of the injected hemispheres and in lateral views of the db-IPS. The hemisphere of case 73 is shown as a right hemisphere. Abbreviations and conventions as in Figures 1, 2; pre-CD indicates the precentral dimple.

**Figure 4.**
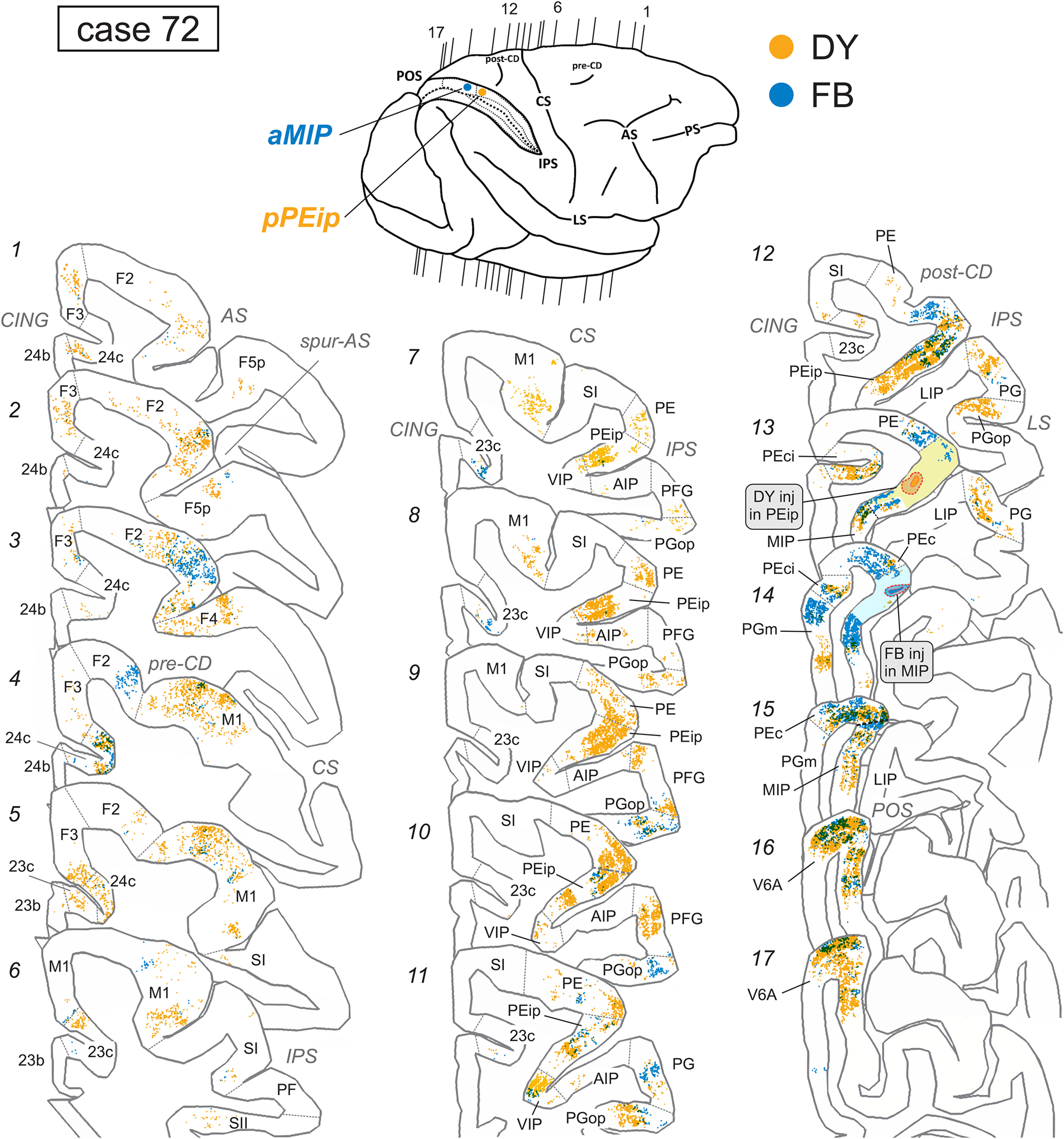
Distribution of retrogradely FB-labeled (blue) and DY-labeled cells (yellow) observed in case 72 after the tracer injections in aMIP and pPEip, respectively, shown in representative sections through the frontal and the parietal cortex. The lightly colored zone surrounding the injection site in sections 13 and 14 corresponds to a sector with homogeneous intrinsic labeling. The levels at which the sections were taken is indicated in the drawing of the hemisphere in the upper part of the figure. POS, parieto-occipital sulcus; post-CD indicates postcentral dimple. Other abbreviations as in Figures 1, 2.

**Figure 5.**
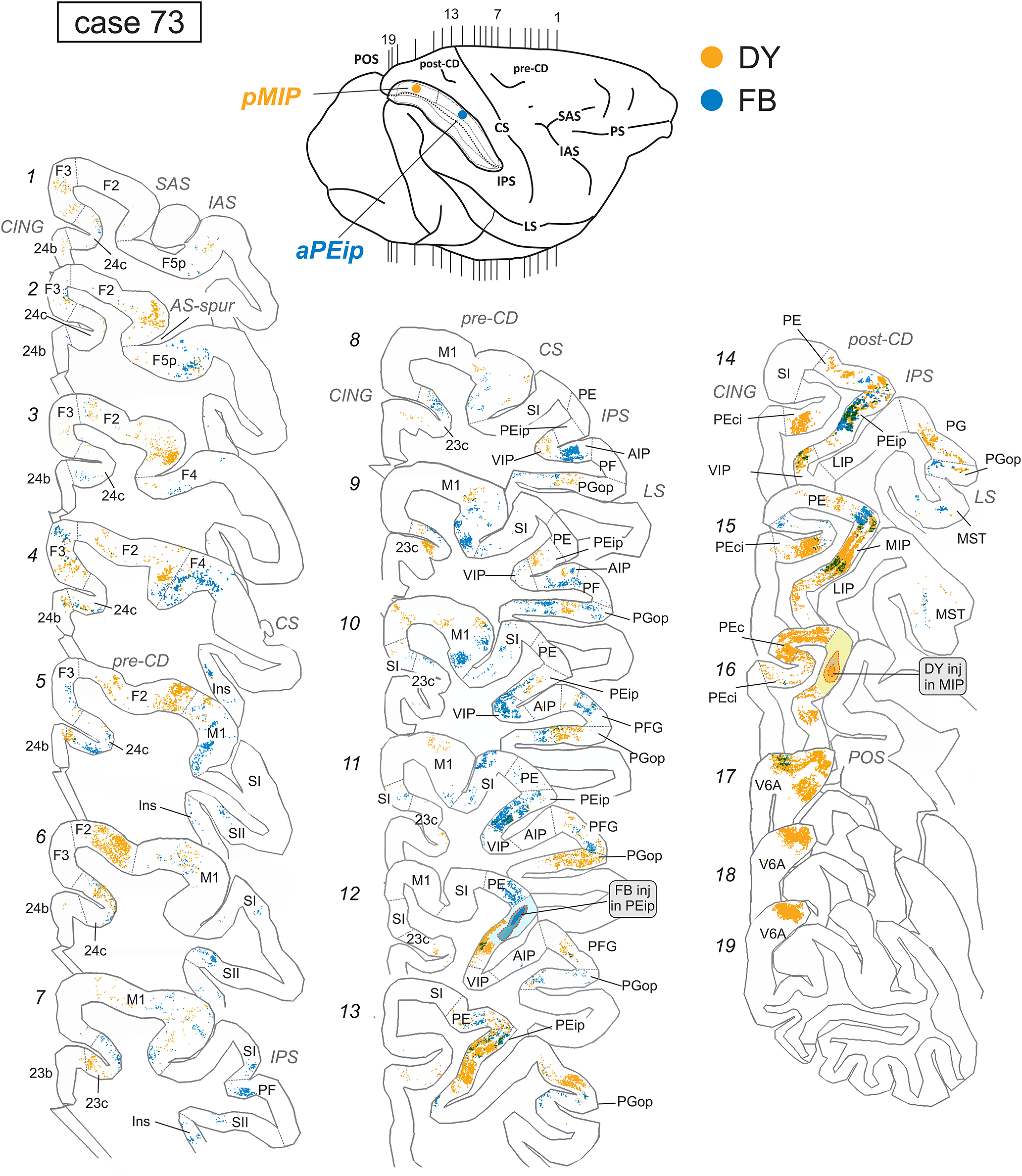
Distribution of retrogradely FB-labeled (blue) and DY-labeled cells (yellow) observed in case 73 after the tracer injections in aPEip and pMIP, respectively, shown in relevant sections through the frontal and the parietal cortex. Conventions and abbreviations as in Figures 1, 2, 4.

Two tracer injections targeted MIP (Fig. 1), one in case 72, where DY was placed in aMIP and one in case 73, where FB was delivered in pMIP. The analysis of the distribution of RLCs in the ipsilateral hemisphere revealed substantial labeling in both frontal and parietal areas with a smaller contribution from selected cingulate zones (Table 1). The results from these two injections will be described together and are illustrated in Figures 3-5.

##### Projections from frontal and cingulate cortex

In frontal cortex, RLCs were found mostly in a region spanning from the ventrorostral sector of area F2 (F2vr), around the spur of the arcuate sulcus, up to the border with primary motor cortex (M1; F1) in the dorsal part of premotor cortex (Figs. 3, 4:*2–4*, 5:*2–6*). In both cases, they represented ∼10% of the total number of RLCs. Labeling extended over the classical arm region described in previous studies that combined anatomic tracing and physiological recording during reaching tasks (Caminiti et al., 1991; Johnson et al., 1996), as well as in the region of the arcuate spur, where neural activity is more related to hand movement (Fogassi et al., 1999). Smaller proportions of RLCs (3.7–3.8%; Figs. 4: *4–6*, 5: *6–11*) were found over the arm region of M1 (see Johnson et al., 1996), lateral to the precentral dimple. No RLCs were found in the mesial part of M1, in the leg and foot representations, in line with data showing that neural activity in MIP is mostly related to visuomotor control of coordinated eye-hand actions.

A very small proportion of RLCs was observed in area F3 [supplementary motor area (SMA); 1.3–1.6%; Fig. 3], and a moderate number of them was located in the agranular cingulate area 24c/d (2–2.7%; Figs. 4: *4–5*, 5: *4–6*) and in the granular cingulate area 23c (1.2–2.3%; Figs. 4: *7–8*, 5: *7–13*).

##### Projections from parietal cortex

In posterior parietal cortex, RLCs were found in both the SPL and, to a lesser extent, IPL. In SPL, after the aMIP injection, there was strong labeling in areas PEc (18.2%; Figs. 3, 4: *14–15*), PEip (17.8%; Figs. 4, 5: *7–13*), and PE (13.8%; Figs. 3, 4: *10–12*), After the pMIP injection, the labeling was similarly robust in PEip (16.5%; Figs. 3, 5: *9–13*), weaker but still strong in PEc (12.5%; Figs. 3, 5: *3–16*), modest in PE (4.1%).

On the medial wall of the SPL, projections from area PEci were stronger to pMIP (12.9%) than to aMIP (6.1%; Figs. 3, 4: *13–14*, 5: *14–16*) and those from PGm were mostly addressed to aMIP (7.1%; Figs. 3, 4: *14*). Finally, projections from area V6A were mostly (22.2%) addressed to pMIP (Fig. 5: *17–19*), but in smaller proportion also to aMIP (7.3%: Fig. 4: *16–17*).

The only IPL areas projecting to MIP, although with a relatively modest proportion of cells (4.3% to pMIP; 3.65% to aMIP), were areas PG (Figs. 3, 4: *11–13*, 5: *13*) and PGop (Figs. 4: *7–12*, 5: *8–13*). RLCs were sparse in VIP (Fig. 4: *7–11*), virtually absent in AIP, absent in LIP. Area MST contained a very small proportion (0.7%) of cells projecting to aMIP. Finally, very few RLCs were observed in SI and SII. No RLCs projection to MIP were found in prefrontal areas.

#### Ipsilateral cortical projections to area PEip

Two tracer injections targeted PEip (Fig. 1), one in case 73, where FB was placed at about its middle part, and one in case 72, where DY was placed in the caudalmost part of it, adjoining the border with MIP (pPEip). As observed after the tracer injections in MIP, RLCs substantially involved frontal and parietal areas, and their distribution reflected A-P gradients of connectivity in the db-IPS.

##### Projections from frontal and cingulate cortex

As shown in Table 1, after both the aPEip and the pPEip injections robust labeling was found in M1 (15.6% and 13.5%, respectively). Robust connectivity with M1, therefore, appears to be a unifying connectional feature of PEip, together with the projection to the spinal cord. In M1, the labeling was mostly located in the medial bank of the CS, thus involving the “new” M1 (Rathelot and Strick, 2009), where hand movements are represented (Figs. 4: *5–8*, 5: *5–10*). After the pPEip injection, RLCs also extended more rostrally in M1 over the cortex of the precentral convexity, lateral to the precentral dimple (pre-CD; Figs. 3, 4: *4–6*). Furthermore, after pPEip, but not aPEip injection, robust labeling was found in F2 (Figs. 3, 4: *1–5*). After the pPEip injection, the proportion of RLCs in F2 (13.4%) was similar to that observed after that in aMIP (10.9%). However, RLCs were almost completely located lateral to the pre-CD, whereas after the MIP injection they extended also more dorsally (Fig. 3). In both cases, moderate labeling also involved the ventral premotor area F4 (Figs. 3, 4: *3*, 5: *3–4*) and weaker labeling was observed in F3 (Figs. 3, 4: *1–3*, 5: *4–5*). Moderate labeling was observed in areas 24c/d and 23c (Figs. 3, 4: *1–7*, 5: *1–8*).

##### Projections from parietal cortex

In the SPL, robust labeling to both aPEip and pPEip was observed in area PE, and it was richer after the aPEip injections (18.3% vs 11.1%). In this area, RLCs very densely packed in the rostral part, however after the pPEip injection they also extended in the caudal part, which was the PE sector densely labeled after the MIP injections (Figs. 3, 4: *7–12*, 5: *11–15*). Caudal to PE, after the pPEip injections, labeling was relatively moderate in PEc (4.2%) and PEci (5.3%), weak in PGm (1.6%), and robust in V6A (10.5%; Figs. 3, 4: *13–17*). In all these areas, labeling was much weaker, or even absent after the aPEip injection (Figs. 3, 5: *14–19*). Similarly, the number of RLCs observed in MIP was much higher after the pPEip (12.9%) than the aPEip (5.1%) injection.

In the IPL, both aPEip and pPEip were moderately connected with the hand-related area PFG, though after the pPEip injection the labeling moderately involved also PG (Figs. 3, 4: *7–13*, 5: *5–7*). Furthermore, aPEip was characterized by a robust input from PGop (11.7%; Fig. 4: *8–10*), which was much weaker for pPEip (4.2%), as well as by relatively robust input from the hand-related area AIP (6.3%) and in VIP (5.5%), where RLCs were relatively sparse after the pPEip injections (Figs. 3, 4: *8–12*, 5: *8–14*).

After the aPEip injection there was robust labeling in SI (7.3%; Figs. 3, 5: *6–7*) and a relatively weak labeling in SII and the insular cortex, all virtually devoid of labeling after the pPEip injection. Finally, a weak input from MST was observed in both cases.

#### Connectivity profiles of aPEip, pPEip, aMIP, and pMIP

**Figure 6.**
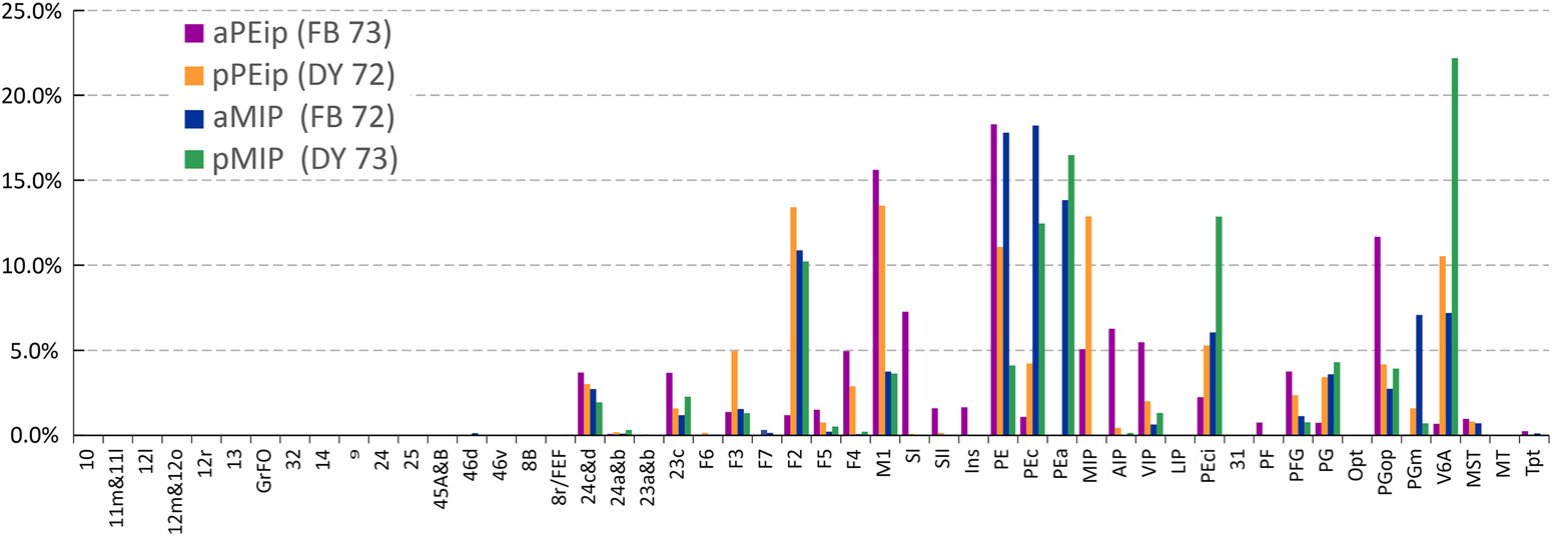
Ipsilateral cortical projections to areas aPEip, pPEip, aMIP, and pMIP. Proportion of cells projecting from different areas to the four injection sites located in area aPEip (purple), pPEip (orange), aMIP (blue), pMIP (green). MIP cells projecting to PEip, and vice versa, are included. Percentages are calculated relative to the total counts of RLCs obtained after each injection.

To offer a quantitative view of the results, the data reported in Figures 4, 5 and in Table 1 were expressed in the form of frequency distribution. Figure 6 reports the proportion of RLCs (*y*-axis) across cortical areas, which are arranged from left to right (*x*-axis) according to their approximate A-P location in the cortex.

The frontal input to parietal areas injected in this study stems mostly from areas F2 and M1. Projections from F2 are mainly addressed to pPEip, aMIP, and pMIP, in decreasing order of magnitude. Motor cortex projections follow a similar pattern but differ for a strong input to aPEip as well. Area SI projects only to aPEip. Smaller projections stem from cingulate areas 24c and 23 and from ventral premotor area F4.

The parietal projections to PEip and MIP are by far stronger that the frontal ones and originate mainly from superior parietal areas, such as PE, PEc, from local connections within PEip and MIP and from V6A, PEci, and PGm. Inferior parietal projections are by far weaker, and originate from PGop, especially after the injection in aPEip, with smaller contribution from areas PG and PFG. Finally, aPEip showed a relatively robust connection with AIP and VIP.

**Figure 7.**
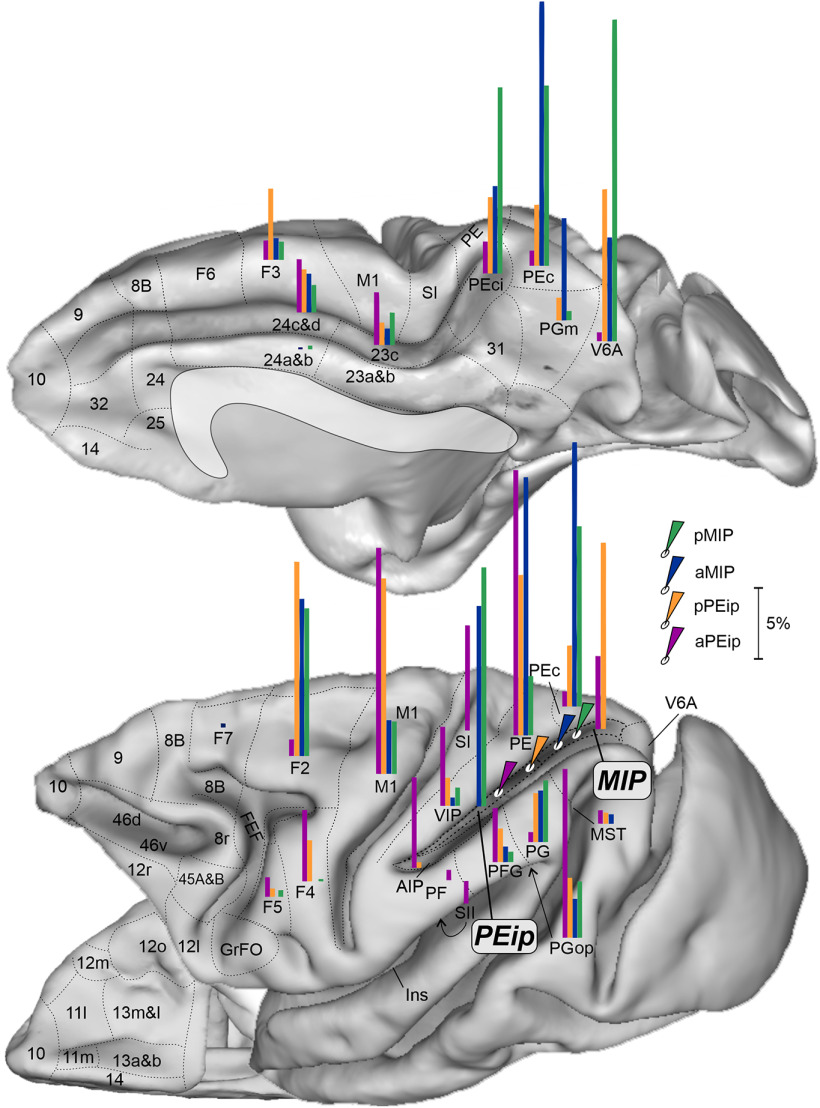
Gradient-like organization of the parietal and frontal projections to the db-IPS. Mesial (top), lateral (bottom, right), and ventral (bottom, left) views of the monkey brain showing the proportion of projecting cells (Fig. 6) in their relative anatomic location, after tracer injections (white ovals with colored arrows) at the four A-P levels of the db-IPS. Each bar has a length proportional to the percent of RLCs (range 1–30%, scale bar corresponding to 5%) to aPEip (purple), pPEip (orange), aMIP (blue), and pMIP (green). Conventions as in previous figures.

In several instances, the projections addressed to PEip and MIP from cingulate, frontal and parietal areas followed a gradient-like pattern, as also shown in Figure 7. Examples are the projections from 24c, M1, and PFG, which all project with decreasing strength to aPEip, pPEip, aMIP, and pMIP. The F2 projections to dorsal intraparietal areas display a similar pattern, if one excludes the scant input to aPEip. On the contrary, the strength of PEci projections shows an inverse gradient. The strength of the projections from PE and V6A waxes and wanes in the A-P extent.

A pictorial representation of the gradient-like organization of this part of the parieto-frontal system can be seen in the brain figurine of Figure 7.

#### Segregation and overlap and laminar distribution of frontal and parietal cells projecting to PEip and MIP

In the tangential domain of the cortex there exists an orderly arrangement of properties that can relate to the representation of sensory receptors, motor output, visual attention, motor intention, working memory, etc. Moreover, there is evidence that cortical connections shape, at least in part, the functional properties of neurons in the parieto-frontal system (Johnson et al., 1996; Chafee and Goldman-Rakic, 1998, 2000; Battaglia-Mayer et al., 2001).

To study whether PEip and MIP share cortical afferents, therefore functional properties, we compared the tangential distribution of frontal and parietal cells projecting to their anterior and posterior sectors, a study made possible by the injections of two different fluorescent tracers in each of the two animals.

In case 72, where DY was injected in pPEip and FB in aMIP, frontal cells projecting mostly to pPEip (Fig. 4, see yellow labeling) involve both dorsal premotor area F2 and M1 while those projecting to aMIP (Fig. 4, see blue labeling) occupy restricted frontal zones, mainly located in F2. With the exclusion of a restricted part of the latter (Fig. 4: *2–3*), cell projecting to pPEip and aMIP were largely segregated in the tangential domain of the cortex. At some locations, parietal cells projecting to both pPEip and aMIP were segregated (Fig. 4: *7–17*), even in the same area, as for PGm (Fig. 4: *14*). On the contrary, extensive overlap was found in areas PEc, PEci, and V6A (Fig. 4: *14–17*).

The distribution of cells projecting to aPEip and pMIP, where FB and DY were, respectively, injected (Fig. 5) obeys to a similar pattern, where segregation dominates over overlap in both frontal and parietal projections, although some overlap was observed in areas PGop (Fig. 5: *10–11*), pPEip (Fig. 5: *13–15*), aMIP (Fig. 5: *15*), and V6a (Fig. 5, 17).

When comparing the distribution of cells in the rostral bank of the CS, i.e., in the new M1 (Rathelot and Strick, 2009), in both cases 72 and 73, we mostly observed absence of overlap of cells projecting to the intraparietal areas injected, as well as in area PE and in large part of aPEip, while a small overlap was confined only to very limited zones of the bank (Fig. 5: *6–7*).

Finally, the analysis of the laminar distribution of RLCs in the various frontal and parietal areas more densely labeled after the injections in different sectors of PEip and MIP showed a proportion of RLCs in the superficial versus deep layers virtually everywhere within 33% and 66%, that is a marked bilaminar distribution.

### DW-MRI study of the db-IPS

#### Comparison between the distribution of RLCs and the diffusion-based connectivity estimates

**Figure 8.**
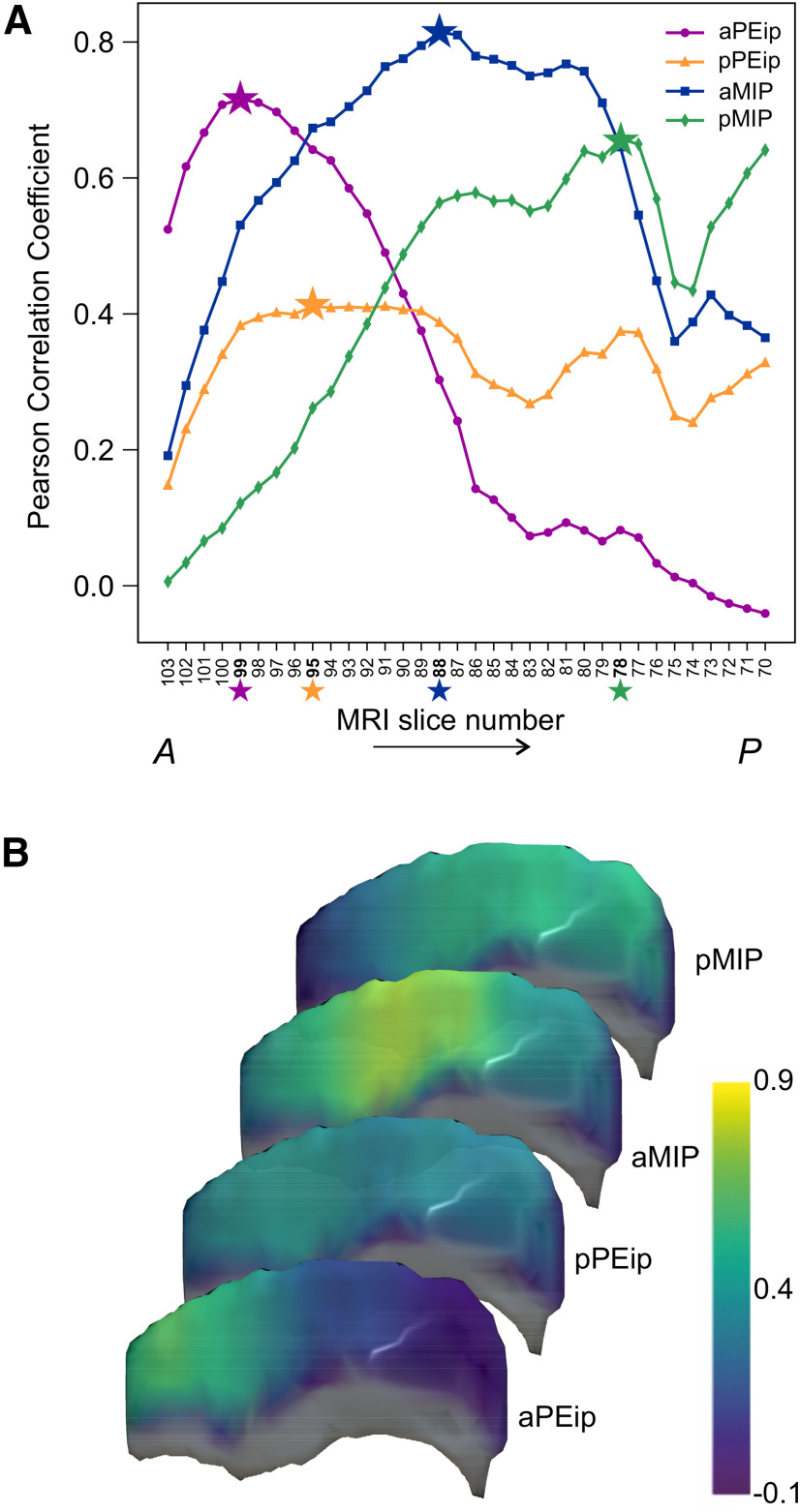
***A***, Pearson’s correlation coefficient between the distribution of diffusion-based connectivity estimated in 2.5-mm windows along the dorsal and middle sectors of the db-IPS and the distribution of labeled cells after the four injections in aPEip, pPEip, aMIP, and pMIP. MRI slice numbers refer to the central position of each sliding window, where slice 103 is the anteriormost and slice 70 the posteriormost. The star markers indicate the A-P location with the highest correlation coefficients. ***B***, The Pearson’s correlation coefficients after each of the four injections are also reported in color code (see bar on the right) across the db-IPS. In this image, the rostralmost part of the db-IPS is not shown, since given its limited D-V extent, it could not be divided into three sectors.

We compared the connectivity of the 48 cortical regions obtained through histologic procedures with the intra-axonal MRI signal fraction estimated from DW-MRI. This was achieved by computing the Pearson’s correlation coefficient between the distribution of RLCs obtained for the four injection sites and the distribution of diffusion-based connectivity estimated at different locations along the entire extent of the db-IPS (see Materials and Methods). To cover in a continuous fashion the whole IPS, we used a sliding window of 2.5 mm, corresponding to five MRI coronal slices, moving in the A-P direction and selecting all streamlines connecting the MRI slices to the 48 cortical ROIs included in our analysis (see Materials and Methods). To better reproduce the extent of the injection sites of retrograde tracers in the D-V dimension, the MRI slices encompassed only the dorsal and middle sectors of our 3-fold subdivision of the db-IPS (Fig. 8*B*). This choice was dictated by the histologic verification that the tracer injections did not involve the deepest part of the dorsal bank, as well as by the fact that the latter can hardly be parcellated into three D-V section in its most anterior part, given the limited extent of the cortex in this dimension.

In Figure 8*A*, data points in each curve show the Pearson’s coefficients for the correlation between the distribution of RLCs obtained for each of the injection sites (aPEip, pPEip, aMIP, pMIP) and the diffusion-based connectivity of each 2.5-mm sliding window along the A-P dimension of the db-IPS. The *x*-axis shows the MRI coronal slice number at the center of each window. The locations with the highest correlation are indicated by the star markers. The MRI coronal slice number corresponding to each injection site’s highest correlation coefficient (Fig. 8*A*, star markers) well agrees with the relative position of the injection sites of neural tracer (Fig. 1). Despite known limitations of DW-MRI connectivity analysis, such as the presence of false-positive connections, Figure 8*A* shows that tractography can indeed identify changes in the connectivity distribution in the A-P dimension of the db-IPS that are correlated with changes observed using RLCs analysis. In fact, the RLCs distribution after injection in aPEip had the highest correlation value (*r* = 0.72, *n* = 34, *p* = 1.1 × 10^−8^) at slice 99, after injection in pPEip at slice 95 (*r* = 041, *n* = 48, *p* = 0.004), showing however similar correlation values (plateau) at different A-P locations ranging from slices 97 to 89, while after injection in aMIP the correlation peaked at slice 88 (*r* = 0.81, *n* = 34, *p* = 1.9 × 10^−12^) and after injection in pMIP at slice 78 (*r* = 0.66, *n* = 34, *p* = 3.9 × 10^−7^). This highlights the sensitivity of the DW-MRI connectivity to the changes measured by the RLCs analysis in the fine parcellation of the db-IPS.

When selecting the locations with highest correlation for each of the four injection sites, the overall correlation between the diffusion-based connectivity estimation and the RLCs distribution was *r* = 0.65 (*n* = 192, *p* = 1.7 × 10^−24^).

**Figure 9.**
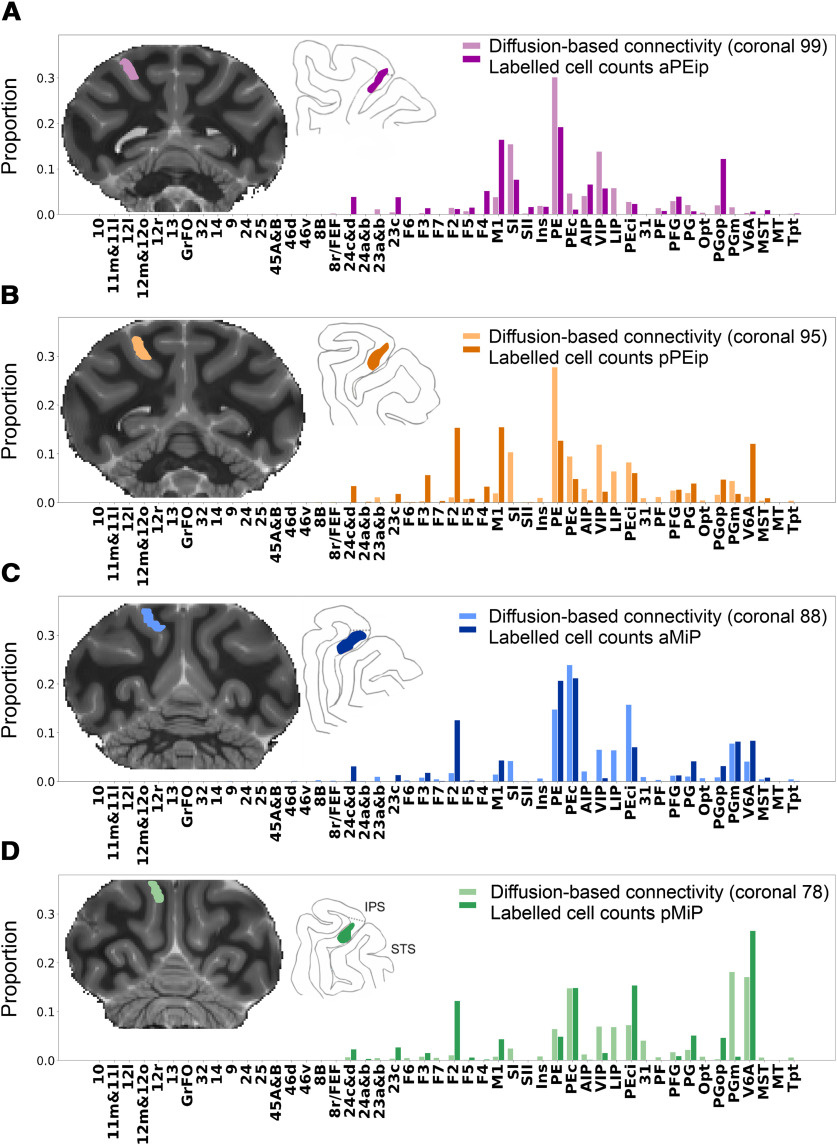
Distribution of labeled cells and diffusion-based connectivity for locations with maximum Pearson’s correlation coefficients (***A***, aPEip: *r* = 0.72; ***B***, pPEip: *r* = 0.41; ***C***, aMIP: *r* = 081; ***D***, pMIP: *r* = 0.66). For each distribution, the MRI slices corresponding to the center positions of the sliding windows with highest Pearson’s correlation coefficients are reported next to the reconstruction of the histologic sections where the injection sites were found. The local connections between MIP and PEip are not reported.

The changes of the correlation coefficient between the distributions of labeled cells and diffusion connectivity across the db-IPS are shown in Figure 8*B*, by using a diffusion MRI derived anatomic rendering of the overall the bank to facilitate the comprehension of the areas involved in this analysis. It can be seen that the highest correlation was found in a region spanning the central part (in A-P dimension) and dorso/middle sectors (in D-V dimension) of the bank, after injections in aMIP. A good correlation was also found in the anterior third of the bank after injections in aPEip, while the correlation decreased, although to a different extent, after injections in pPEip and pMIP. The implication of these results for the gradients in the connectivity profiles of the dorsal intraparietal areas will be dealt with in the Discussion. The corresponding distribution of RLCs for the four injection sites alongside the diffusion-based connectivity for the locations with the maximum Pearson’s coefficients are reported in Figure 9, together with the relative MRI slices and drawing of the histologic sections.

For the four injections sites there are 192 (48 areas × four injections) potential ROIs connections, among which 113 have non-zero labeled cell counts. Diffusion tractography shows an average of 90.4% of the connection’s weights for ROIs with non-zero reported labeled cells. Moreover, tractography correctly identified 107 connections (true-positive connections; TP), thus missing only five connections (false-negative connection; FN). Tractography correctly reported no connectivity for 44 ROIs (true negative connections; TN), but estimated connectivity for 36 ROIs where no labeled cells were found (false positive connections; FP).

The overall data analysis resulted in a sensitivity of 0.96 $(\frac{TP}{TP+FN})$ and a specificity of 0.55 $(\frac{TN}{TN+FP})$.

Across the matching locations and all cortical ROIs, the connection with the most underestimated fraction of diffusion-based connectivity (−0.143) is ROI F2, after injection site in aMIP. This is followed by connection F1-aMIP (−0.135), F1-pMIP (−0.126), F2-aPEip (−0.111), and F2-pPEip (−0.109). Similarly, the most overestimated connectivity is PGm-aPEip (+0.173), followed by PE-aMIP (+0.151), PE-pMIP (+0.109), SI-aMIP (+0.103), and VIP-aMIP (+0.096). Across the four matching site’s location, tractography misestimated the connectivity the most on ROIs F2, PE, M1, VIP and LIP. As examples, contrary to tracer data, our tractography estimations showed streamlines connecting both sectors of PEip (Fig. 9*A*,*B*) and MIP (Fig. 9*C*,*D*) to LIP. However, previous histologic studies had shown connections between LIP and MIP (Bakola et al., 2017) and LIP and PEip (referred to as PEa; Blatt et al., 1990). Furthermore, our study shows connections between aPEip and SI (Fig. 9*A*) which are stronger from tractography than histology. It also reveals streamlines between SI and pPEip (Fig. 9*B*) and both sectors of MIP (Fig. 9*C*,*D*), which are not matched by histology (see also Table 1). Finally, cell counts show strong connectivity between F2 and pPEip (Fig. 9*B*), as well as with and both sectors of MIP (Fig. 9*C*,*D*), which is not matched by tractography.

#### Diffusion-based connectivity profiles along the db-IPS

**Figure 10.**
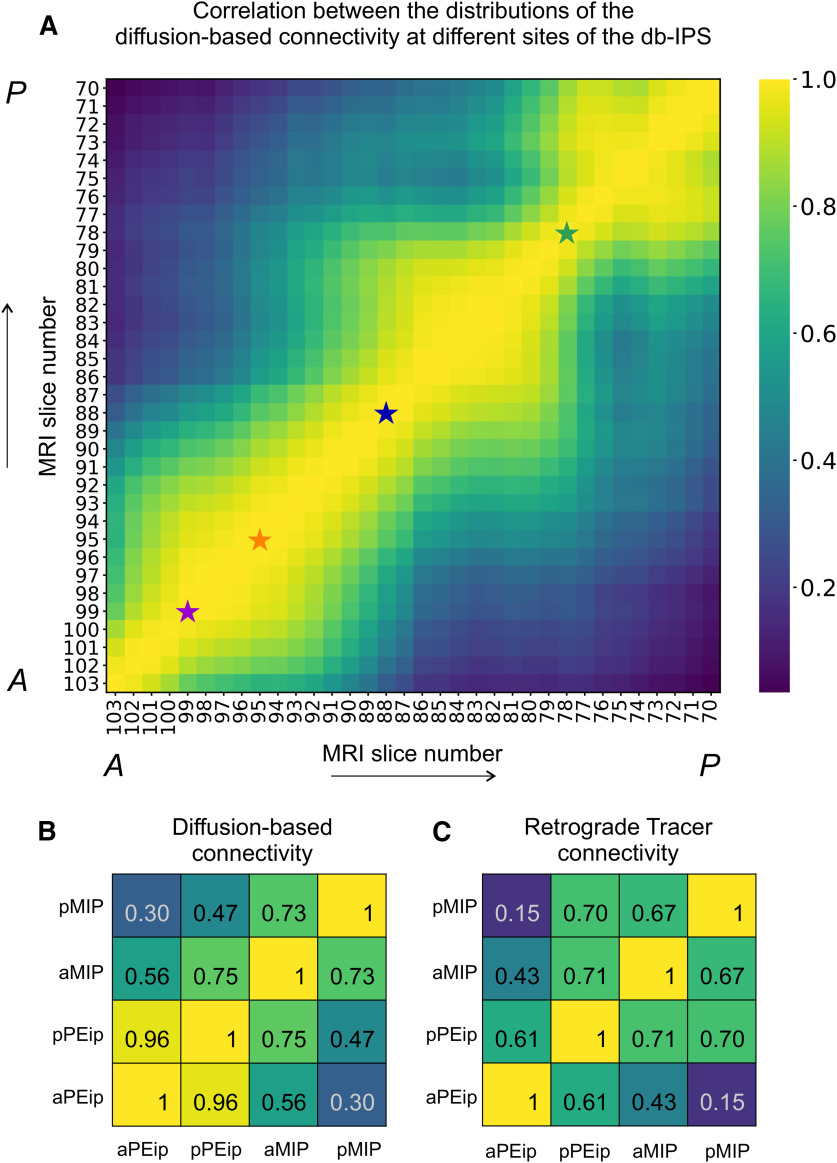
***A***, Pearson’s correlation coefficient between the distributions of the diffusion-based connectivity estimated in subregions along the db-IPS, as defined by a sliding window of 2.5 mm moving in the A-P direction (five MRI coronal slices). For each window, the connectivity is evaluated first by selecting all the streamlines connecting the MRI slices to the 48 ROIs included in the analysis and summing the contribution to the intra-axonal MRI signal fraction of each streamline for each cortical area. Data were normalized relative to the total contribution of the streamlines associated to each sliding window. The *x*- and *y*-axes show the MRI slice number corresponding to center position of each window. Star markers (slices 99, 95, 88, and 78) indicate the locations with highest correlation coefficient between diffusion-based connectivity and labeled cells, after tracer injections in aPEip, pPEip, aMIP, and pMIP (Fig. 8). Values of correlation coefficients are indicated by the color code (see bar on the right). ***B***, Pearson’s correlation coefficients between the distributions of diffusion-based connectivity estimated at the four sites reported above. **C.** Pearson’s correlation coefficients between the distributions of RLCs after injection in aPEip, pPEip, aMIP, and pMIP. In ***B***, ***C*** correlation coefficients are also reported with relative values (color code as in ***A***).

As a next step, we evaluated in a quantitative fashion the degree of similarity of the diffusion-based connectivity estimation along the db-IPS. Figure 10*A* shows the Pearson’s correlation coefficient between the distributions of diffusion-based connectivity estimated in different sliding windows along the A-P extent of the db-IPS. The *x*- and *y*-axes show the MRI coronal slice number corresponding to the center of each window. A strong correlation is expected between locations distant four or less MRI slices apart, because of the windows overlap. A decrease in correlation can be observed when the distance between windows increases in the A-P extent of the bank. This suggests a general gradient-like organization, where the pattern of cortical connectivity gradually changes. Visual inspection of the correlation matrix highlights the existence of three potential clusters, located anteriorly, centrally, and posteriorly along the bank, that can be identified by their highest correlations (range 1–0.6) between neighboring locations. This suggests that along the A-P extent of the db-IPS there might exist three broad hodologically different regions. A similar matrix (Fig. 10*B*) is shown for selected locations corresponding to the four MRI windows with the highest correlation between the diffusion-based and tract tracing connectivity (see also Fig. 8). It can be seen that similar results were obtained when correlating the pattern of connectivity obtained from histologic tracing data, after injections in intraparietal areas aPEip, pPEip, aMIP, and pMIP.

#### DW-MRI connectivity estimates of the dorsal, middle, and ventral sectors of the db-IPS

**Figure 11.**
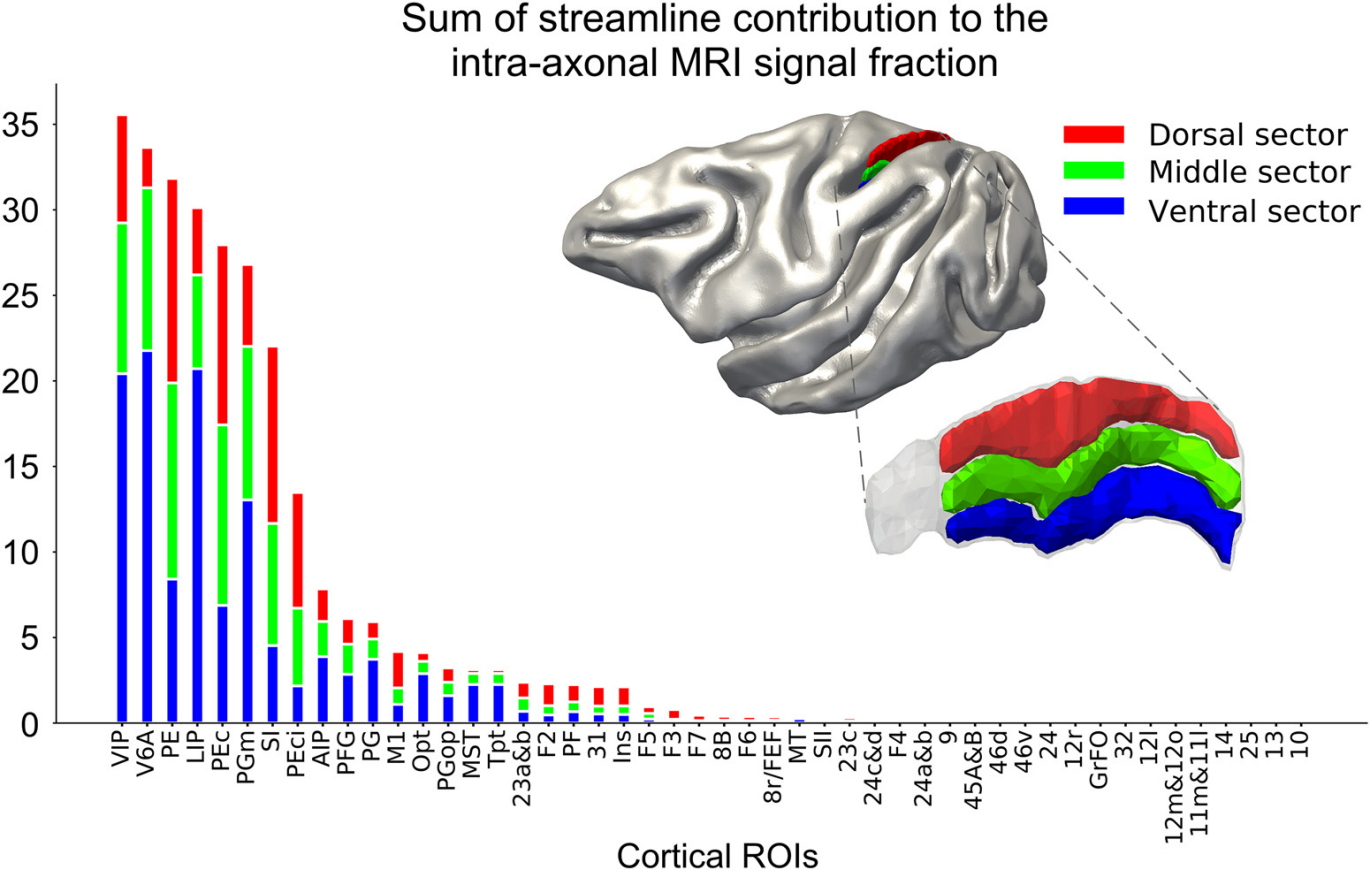
Sum of the cortical connectivity of the db-IPS to other cortical ROIs. For each ROI, the diffusion-based connectivity estimation is reported for the dorsal (red), middle (green), and ventral (blue) sectors. The diffusion connectivity corresponds to the sum of streamline contributions to the intra-axonal MRI signal fraction estimated using COMMIT for each cortical ROI. The sectors of the db-IPS are shown on the mid cortical surface (top right) and on the db-IPS (bottom right). Notice that the rostralmost part of the db-IPS (gray region) was not used for this analysis, since it could not be parcellated intro three D-V sectors, given its limited extent in the D-V dimension).

Furthermore, we investigated the cortical connectivity of the dorsal, middle and ventral sectors of the db-IPS using diffusion MRI. It is worth stressing, the cortical regions lying in the more ventral and deep part of the bank can be hardly accessed by neural tracer injections, therefore their connectivity remains virtually unknown. The sum of the diffusion-based connectivity calculated across the 38 different A-P locations (MRI slices) for the dorsal, middle and ventral sectors is shown in Figure 11. The parietal areas VIP, V6A, PE, LIP, PEc, PGm, and SI are the ROIs showing the overall strongest connectivity with the bank, among the 48 ROIs considered in this study. However, clear differences emerge in the streamline contribution provided by specific portions of the IPS along the D-V dimension.

**Figure 12.**
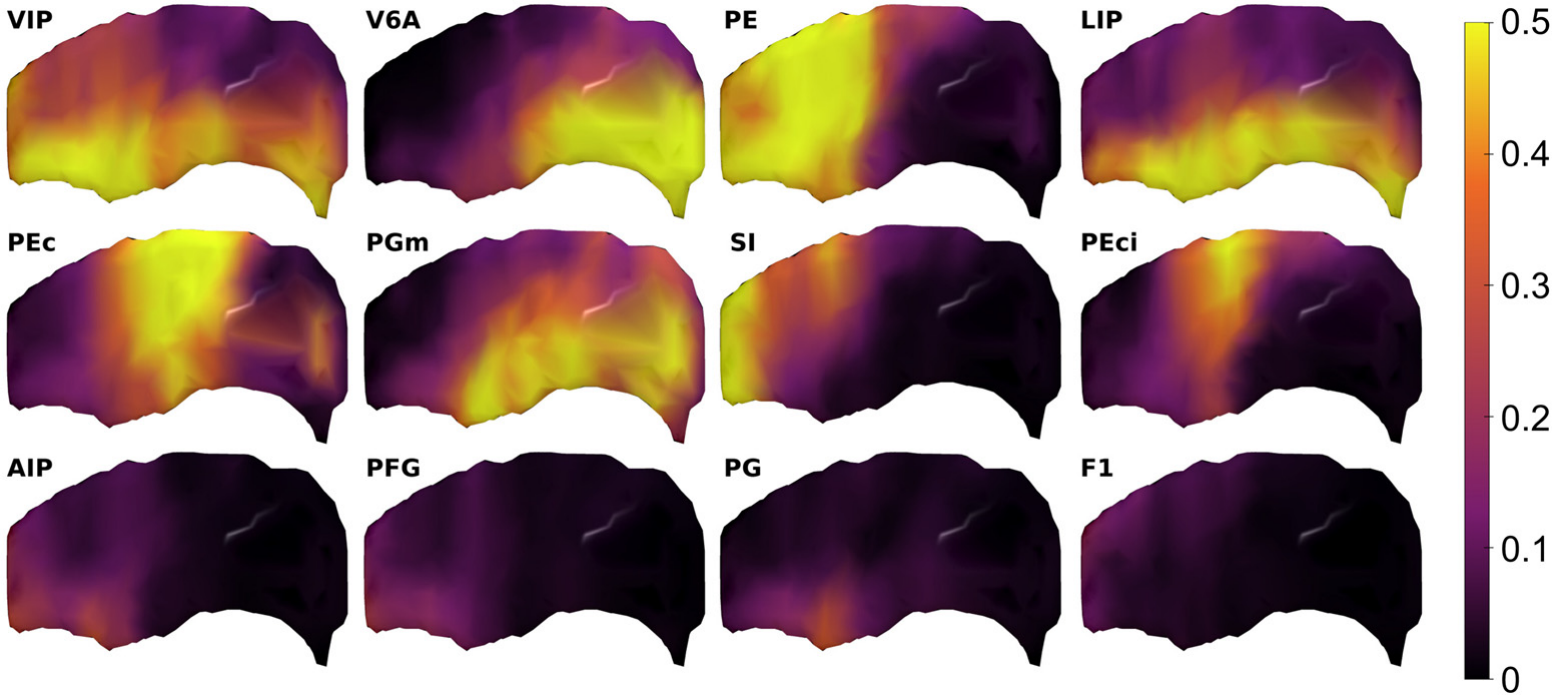
Spatial distribution of the IPS connectivity estimated from DW-MRI along 34 dorsal, middle, and ventral A-P sectors of the db-IPS, for the 12 cortical ROIs displaying the strongest estimated connectivity with the db-IPS (Fig. 11). The yellow and orange regions highlight the IPS locations with the strongest estimated connectivity for the corresponding cortical areas.

To highlight this aspect, we report the results (Fig. 12) referring to the connectivity occurring between each of the 12 most connected cortical areas (i.e., VIP, V6A, PE, LIP, PEc, PGm, SI, PEci, AIP, PFG, PG, M1; see Fig. 11), and the A-P and D-V extent of the db-IPS. Each image shows the spatial distribution of the diffusion-based connectivity, along the 38 A-P dorsal, middle and ventral subdivisions of the bank, for each of the 12 cortical ROIs listed above. The sectors displaying strong connectivity with the indicated cortical ROI are shown in yellow and orange. It can be seen that there exists a smooth transition in the strength of connectivity in both the A-P and D-V dimensions of the bank. The IPS region more strongly connected with area VIP is the most anterior sector of the bank, with a gradual reduction moving posteriorly, while for V6A is the posteroventral part of the bank, as also observed from tract tracing data on the proportion of RLCs (Fig. 7). Area PE instead display a more diffuse pattern of connectivity along the D-V dimension of the anterior part of the bank. LIP connectivity occurs exclusively with the regions located in the more ventral part of the dorsal bank, close to the fundus of the IPS. Another example of a gradient-like distribution of connectivity, along both the A-P and D-V dimensions is offered by PEc, whose connectivity is strongest with the dorsal and intermediate part of the bank. The connectivity of PGm resembles that of V6A, but it is weaker and more diffuse in the A-P extent of the ventral part of the intermediate sectors. Area SI is strongly connected with the D-V extent of the rostralmost part of the bank, while the connections of PEci are more selective, since they occur mainly with the central part of the bank, are stronger dorsally and fade away moving ventrally, anteriorly and posteriorly. The inferior parietal areas AIP, PFG, and PG show a weak connectivity with the anterior part of the ventral sector of the bank, while motor cortex (M1) is weakly connected with its anterodorsal sector.

## Discussion

The results of this study provide solid support for a parcellation of the db-IPS into a rostral area PEip and a caudal area MIP, based on corticospinal projections, as well as for an internal subdivision of both areas into an anterior and posterior sector. Our data also show A-P and D-V connectional gradients, matching those of functional properties described by electrophysiological studies. In the A-P extent of the SPL Crammond and Kalaska (1989) and Burbaud et al. (1991) showed that activity in area PE is mostly related to somatosensory and somatomotor functions, while Colby and Duhamel (1991) in MIP described a set visuomotor functions. A combined anatomo-functional analysis of the parieto-frontal system (Johnson et al., 1996) in monkeys revealed that reaching-related neurons displaying signal-related, set-related, movement-related, and positional-related activity decreased in numbers moving from ventral to dorsal in MIP, up to area PE. A similar trend was found in the A-P dimension of the frontal lobe, from F7 to F2 and MI. Furthermore, parietal and frontal regions displaying similar activity types were linked by direct corticocortical connections.

### Cortical connections of the db-IPS

Our data are in line but also extend data from Bakola et al. (2017), where MIP defined myeloarchitectonically extends rostrally up to the A-P level of the caudal end of the central sulcus, thus including the caudalmost part of the corticospinal sector of the db-IPS.

Our tracer injections in MIP show a relatively strong connectivity with visuomotor areas V6A, PEc, PEip, and F2. Weaker connections involve the IPL visuomotor area PG, area PGop and M1. Furthermore, aMIP, when compared with pMIP, shows stronger connectivity with area PE and visuomotor area PGm, a weaker one with somatosensory-related area PEci. This connectivity pattern of MIP conforms to that reported by Bakola et al. (2017) for the caudal part of this area. Furthermore, indirect support for this connectivity scheme and for the reciprocity characterizing MIP connections comes from studies in which this area was labeled after retrograde tracer injections in V6A (Marconi et al., 2001; Gamberini et al., 2009; Passarelli et al., 2011), PEc and PE (Marconi et al., 2001; Bakola et al., 2010, 2013), PGm (Passarelli et al., 2018), PG (Rozzi et al., 2006), and F2 (Johnson et al., 1996; Matelli et al., 1998; Marconi et al., 2001; Tanné-Gariépy et al., 2002). Thus, the connectivity of MIP provides a neural substrate for the visuomotor control of reaching and eye-hand coordination, since it can serve as interface between the premotor areas of the frontal lobe and the parieto-occipital areas V6A and PEc, where neurons combine in a directionally-congruent fashion eye-related and hand-related positional and movement signals within their directional tuning fields (Battaglia-Mayer et al., 2000, 2001). Interestingly, similar inputs to MIP come from PGm (7m), where individual neurons also integrate visual, eye and hand information (Ferraina et al., 1997a,b).

A model relevant to eye-hand coordination (Mascaro et al., 2003) integrating inputs from the retinal position of the target with eye and hand position shows that both feedforward and recurrent interactions of these signals account very well for the experimentally observed tuning fields of parietal neurons. In this model, the representation of directional variables concerning hand and eye movement emerges from Hebbian synaptic plasticity alone (see Battaglia-Mayer and Caminiti, 2002, 2018; Battaglia-Mayer et al., 2015).

Our data also show that area PEip displays as unifying connectional features a robust connectivity with the cervical spinal cord and the hand field of M1. Strong connections with area PE and with visuomotor hand-related area PFG (Ferrari-Toniolo et al., 2015), bimodal visual and somatosensory area VIP, and area F4 further characterize PEip. The caudal part of PEip also displays connections with V6A and F2 and a connectivity pattern with areas PEci, PEc, and PG quantitatively similar to that of aMIP. In contrast, aPEip displays connections with the arm/hand field of SI, the hand-related area AIP and a strong link with PGop, whose function remains unknown. The connectivity observed after tracer injections in pPEip and aPEip is very similar to that observed by Bakola et al. (2017) after an injection in rostral myeloarchitectonic area MIP and in area PEip, respectively. Connections with PEip have been observed after retrograde tracer injections in areas V6A (Gamberini et al., 2009), PE (Bakola et al., 2013), PFG (Rozzi et al., 2006), AIP (Borra et al., 2008; Lanzilotto et al., 2019), F2 (Johnson et al., 1996; Matelli et al., 1998; Tanné-Gariépy et al., 2002), and M1 (Strick and Kim, 1978; Matelli et al., 1986; Hatanaka et al., 2001). This connectivity suggests a role of PEip in sensorimotor control of hand movements. Indeed, PEip as a whole coincides with the db-IPS sector hosting corticospinal neurons projecting to distal hand muscles motorneurons (Rathelot et al., 2017), as well as neurons with somatosensory receptive fields on the hand (Iwamura et al., 1994; Iwamura, 2000; Seelke et al., 2012). The posterior part of PEip could also correspond to the sector hosting neurons with bimodal, visual and somatosensory receptive field centered on the hand (Iriki et al., 1996) while anterior PEip to the sector rich in grasping-related neurons (Gardner et al., 2007). The connectional differences between the posterior and the anterior part of PEip, suggest for the former a role in visuomotor and somatomotor control of hand and arm movements, and for the latter a role in somatomotor control of hand actions.

### Diffusion-based connectivity estimations

We have used state-of-the-art tractography algorithm and microstructure method to estimate the intra-axonal MRI signal fraction associated with streamlines, instead of using their number. This reduced density biases associated with white matter bundle features, such as length, curvature, and size, making tractography more quantitative (Girard et al., 2014; Daducci, et al., 2015). This goal was achieved by using a model of tissue microstructure (Stick–Zeppelin–Ball model; Panagiotaki et al., 2012; Daducci et al., 2015) to explain the measured DW-MRI signal from the streamlines, by removing or penalizing redundant or inaccurate trajectories. In a previous study, Girard et al. (2020) compared various diffusion-based connectivity estimation approaches in the monkey brain and showed that this model had strong performances in the prediction of parieto-frontal binary connectivity (sensitivity and specificity). Moreover, the model provided the highest fraction of valid connectivity weight among methods with high sensitivity and specificity.

In the connectivity network emerging after the four injections made within the db-IPS, our results showed an increased sensitivity of 0.96 (from 0.79) and a decreased specificity of 0.55 (from 0.60), as compared with the analysis of the parieto-frontal network we made before (Girard et al., 2020). Overall, this resulted in an increased Youden’s index (sensitivity + specificity – 1; Youden, 1950) to 0.51 versus the 0.39 reported in Girard et al. (2020). Moreover, in the network studied here, we found 90.4% of the connectivity weights between ROIs with reported non-zero labeled cell count, 10.2% more than in Girard et al. (2020). This suggests a strong predictive power of tractography for the connectivity of the monkeys IPS, which was also confirmed by the lack of connections with prefrontal areas shown by both histologic and tractography results.

In addition to the rostro-caudal gradients evidenced by the tracer injections, the tractography estimated connectivity showed along the db-IPS clear D-V gradients which would have been difficult to demonstrate based on tracer injections. These consisted in a preferential connectivity of ventral sectors of the bank with visuomotor areas V6A, PGm, and LIP and a preferential connectivity of middle and dorsal sectors with SI, PE, PEci, PEc, thus matching the increase in visually responsive neurons moving from dorsal to ventral in the bank (Colby and Duhamel, 1991; Johnson et al., 1996; see Battaglia-Mayer et al., 2016). D-V chemoarchitectonic differences within the db-IPS, waiting for functional and/or connectional correlation, have been observed based on receptor autoradiography (Niu et al., 2020).

Our overall correlation of the diffusion-based connectivity and of the RLCs distribution (*r* = 0.65) goes in line with the results (*r* = 0.59) reported by Donahue et al. (2016). These authors studied the predictive power of tractography for connection weights derived from 29 retrograde tracer injections and 91 areas, reported by Markov et al. (2014). Although we have used different tractography algorithms and connectivity weights estimation from DW-MRI, both Donahue et al. (2016) and our study show that tractography can indeed estimate structural connectivity weights correlated with the number of measured labeled cells connecting cortical areas.

### Tractography misestimated connections

Although tractography produces weighted connectivity proportions showing a good correlation with the proportions of labeled cells, and that most of the weights are in connections with non-zero measured labeled cell count, some connection weights were misestimated.

The source of these misestimations can be related, in part, to the unidirectional labeling of cells from retrograde axonal tracing used in this study. Thus, asymmetry in the afferent and efferent axon densities of a fascicle could result in a mismatch between the two techniques. The diffusion-based connectivity was estimated from ROIs in the db-IPS that were larger than the injection site of tracers, thus reporting the connectivity of a broader sector. Moreover, the intricate white matter geometries and configurations, such as crossing and kissing, could have generated incorrect orientations and erroneous trajectories (Jeurissen et al., 2019; Girard et al., 2020). Finally, the accuracy of diffusion-based connectivity is limited by the model of the white matter used, which can fail to accurately model the diffusion signal in intricate microstructure environments (Jelescu et al., 2020). Future work should target analysis of ROIs with misestimated connectivity, using DW-MRI and bidirectional tracing data of the same animal.

## Synthesis

Reviewing Editor: Nicholas J. Priebe, University of Texas at Austin Decisions are customarily a result of the Reviewing Editor and the peer reviewers coming together and discussing their recommendations until a consensus is reached. When revisions are invited, a fact-based synthesis statement explaining their decision and outlining what is needed to prepare a revision will be listed below. The following reviewer(s) agreed to reveal their identity: Nicole Eichert, Nicola Palomero-Gallagher. Note: If this manuscript was transferred from JNeurosci and a decision was made to accept the manuscript without peer review, a brief statement to this effect will instead be what is listed below.

SYNOPSIS

Two reviewers evaluated the manuscript “The Complex Hodological Architecture of the Macaque Dorsal Intraparietal Areas as Emerging from Neural Tracers and DW-MRI Tractography” and both reviewers agreed that the manuscript makes important connectivity measures for the areas near the intraparietal sulcus. The reviewers appreciated that the manuscript contains direct tracing experiments as well as connectivity measures based on diffusion imaging. While the reviewers recognized the importance of this work, there was consensus that two major issues that should be addressed in a revision of the document. The first issue is that the manuscript should provide a basis for the cyctoarchitectonic definition of the areas, and it should also include a discussion of how those divisions in the present manuscript are related to the previous literature. The second issue that arose is that the present manuscript lacks key details related to the quantification of the data. Those details are enumerated in the comments from Reviewer #1. Both the reviewers also suggest that the visual abstract would be better without the lower colormaps. The reviewers provided detailed suggestions which you should find useful in revising your manuscript, if you decide to resubmit it to eNeuro or to another journal.

-----------

REVIEWER #1

ADVANCES THE FIELD

This manuscript represents a valuable contribution to our understanding of the connectivity of the intra-parietal sulcus. In particular, the authors compare connectivity estimates obtained from retrograde tracing experiments and diffusion MRI modelling, which validates and extends their findings.

COMMENTS

This manuscript represents a valuable contribution to our understanding of the connectivity of the intra-parietal sulcus. In particular, the authors compare connectivity estimates obtained from retrograde tracing experiments and diffusion MRI modelling, which validates and extends their findings. Overall, the rationale and structure of their argument is clear, but the well-written introduction and discussion are somehow contrasted by oversights and lack of clarity in the Methods and Results section. In a revised version of the manuscript, the authors should improve the description of the quantifications and the figures. Please find below my detailed list of comments and suggestions to improve the paper. Furthermore, I’d like to encourage the authors to make the raw-values underlying their presented quantifications available to the reader.

- l. 112.: introduce the abbreviation anterior-posterior

- l. 135: Please consider splitting up the individual sections within the Material and Methods so that different aspects are described under the relevant subheading and not in one continuous block (for example ‘Subjects’ and ‘Surgical procedures’, etc.)

- l. 161. ‘Tracer injection’: Please include the location abbreviation (aMIP, etc) in the body of the text, so that it can be more easily identified in the Figure legend

- l. 189 Is the figure showing the injection sites for Case 10 and 21 missing? If there is no such figure, please place the reference to Figure 1 further above with the description of Case 22/23.

- Figure 1: I suggest placing the 3D rendering of the brain below Case 73 and label with something like ‘summary’. Otherwise one might try to relate it to Case 72. Please specify the hemisphere that was injected for each case in the figure legend. The figurines imply that they were injected in the same hemisphere, but the body of the text describes the opposite. The caption ends with an incomplete sentence

- l. 220. Please introduce RLC. I’m assuming retrogradely-labelled cells.

- l. 222. Which computer-based charting system? Please provide the software name or were in-house scripts used?

- l. 280: Please clarify: Do the segments ‘dorsal, middle, ventral’ correspond to ‘dorsal bank, fundus of the sulcus and ventral bank’? Or is this a segmentation only of the dorsal bank?

- l. 311: “ [...] normalizing the sum of the COMMIT weights of each ROI by the sum of COMMIT weights of all ROIs of this sliding window”. Does this only refer to dorsal/middle vs ventral segment? Or all other cortical ROIs? The entire paragraph is difficult to parse, please consider rephrasing in simpler words.

- Figure 2: were the red dots annotated manually or using the ‘computer-based charting system’ (see above)? Some details on the definition of RCLs seems to be missing in the methods section.

- Figure 3: pre-CD is not labeled in the figure but referred to several times in the main text. Please consider adding a label.

- l. 441: Figure 7, should be Figure 6, I think. l. 457: Fig. 8 should be Figure 7, I think etc, Figure numbers flipped several times, please update.

- l. 518. It is noted that only dorsal and middle sectors are used, but further above it says that dorsal and middle segment were merged (l. 304). This seems inconsistent, please clarify

-l. 506. ‘the correlation between the distribution of RLC obtained after each injection sites and the distribution of diffusion-based connectivity [etc...]’. I found this sentence (and also the next) hard to understand and given that this is a key metric it should be rephrased so it is clear which numbers were correlated. It only became clear to me when looking at Figure 9

- is it not somehow an artifact that the correlation is maximal at the injection site? Please describe the rational why this is an interesting finding and why this is not expected a priori.

- Figure 8B: Please describe more clearly what is shown. The figure displays a 3D rendering of the dorsal bank of the db-IPS, but on a first glance I mistook it for an inflated brain surface. I’m not convinced this sub-figure is adding much compared to Figure 8A the way it is currently described in the main body.

- Figure 9: A colour legend is missing. I would also suggest to use a different colour mapping as it’s not clear why both pPEip and aMIP are shown in blue.

- Figure 12. Could a similar summary figure be constructed for the tract-tracing data? This would visualize the finding that the two modalities produce results that are in part overlapping, but also diverge.

-----------

REVIEWER #2

ADVANCES THE FIELD

This combined histological and MRI diffusion based tractography approach constitutes a valuable resource for the community, since it provides a gold standard cross-validation of the relationship between proportion of labelled cells projecting to PEip and MIP, and measures of diffusion tractography connectivity. Furthermore, the authors demonstrate a significant correlation between the proportion of cells projecting to MIP and/or PEip, and the diffusion-based connectivity estimates of the corresponding streamlines.

COMMENTS

It is not clear to me how authors identified the cytoarchitectonic areas described in the results section as containing retrogradely labelled cells. A histological cell body staining? Neu-N immunohistochemistry? I did not find this information in the section dedicated to histological procedures and data analysis. Furthermore, although PEip and MIP can be distinguished based on their corticospinal connectivity, the same does not apply to their rostrocaudal subdivisions. Thus figures depicting their cytoarchitecture to confirm identification would be helpful. The same applies to areas with retrogradely labelled neurons.

How do the connectivity patterns depicted in Figure 12 correlate with cytoarchitectonically identified areas? The authors should delineate their cytoarchitectonically identified areas - or provide an additional panel with a schematic representation. Do their findings reconcile the differences between the map of Pandya and Seltzer (1982) and that of Niu et al. (2020)?

Minor points

The legend to figure 1 is incomplete. This also makes interpretation of Figs 2 and 3 a bit difficult, as they refer to the abbreviation list of Fig. 1.

Figure 12 would benefit from the labelling of areas within the region of interest

There are some typos:

- Line 154, “Oxygen/ Isoflurane” should be “Oxygen/Isoflurane"

- Line 393, “very few. RLC were observed” should be “very few RLC were observed"

- Line 1064, “db_IPS” should be “db-IPS

## Author Response

We are very grateful to the reviewers for their detailed and useful comments to the manuscript. We have

accepted all of them and changed (in red) the text accordingly. Please see below our point-to point reply.

REVIEWER #1

ADVANCES THE FIELD This manuscript represents a valuable contribution to our understanding of the

connectivity of the intra-parietal sulcus. In particular, the authors compare connectivity estimates obtained

from retrograde tracing experiments and diffusion MRI modelling, which validates and extends their

findings.

COMMENTS This manuscript represents a valuable contribution to our understanding of the connectivity of

the intra-parietal sulcus. In particular, the authors compare connectivity estimates obtained from

retrograde tracing experiments and diffusion MRI modelling, which validates and extends their findings.

Overall, the rationale and structure of their argument is clear, but the well-written introduction and

discussion are somehow contrasted by oversights and lack of clarity in the Methods and Results section. In

a revised version of the manuscript, the authors should improve the description of the quantifications and

the figures. Please find below my detailed list of comments and suggestions to improve the paper.

Furthermore, I’d like to encourage the authors to make the raw-values underlying their presented

quantifications available to the reader.

l. 112.: introduce the abbreviation anterior-posterior

Reply: this has been done, please see pag. 5

- l. 135: Please consider splitting up the individual sections within the Material and Methods so that

different aspects are described under the relevant subheading and not in one continuous block (for

example ‘Subjects’ and ‘Surgical procedures’, etc.)

Reply: Thank you, very good suggestion, this has been done, please see pag. 4 and following in Methods

section

l. 161. ‘Tracer injection’: Please include the location abbreviation (aMIP, etc) in the body of the text, so that

it can be more easily identified in the Figure legend -

Reply: this has been done in the Introduction, please see pag. 2.

l. 189. Is the figure showing the injection sites for Case 10 and 21 missing? If there is no such figure, please

place the reference to Figure 1 further above with the description of Case 22/23. I suggest placing the 3D

rendering of the brain below Case 73 and label with something like ‘summary’. Otherwise one might try to

relate it to Case 72.

Reply: Thank you for noticing this incongruency. Cases 72/73 are shown in Fig. 1, but the problem the

referee noticed probably depended on a non-optimal organization of the figure, that now has been

rearranged as to make the injection sites of cases 72/73 well in evidence. Please see new version of Fig. 1.

Furthermore, in Figure 2 we have added coronal sections through the spinal cord showing the location of

the injection sites.

Please specify the hemisphere that was injected for each case in the figure legend. The figurines imply that

they were injected in the same hemisphere, but the body of the text describes the opposite.

Reply: Tracers were injected in the right hemisphere in case 72 and in the left hemisphere in case 73.

However, to facilitate inspection of data and comparison between the results, data are represented as if 2

they were obtained in both cases from the right hemisphere. This is now specified in the legend of Fig. 1 and

in the text, please see pag. 5.

The caption ends with an incomplete sentence

Reply: We are sorry for not controlling carefully the .pdf, the caption was complete in the Word version of

the ms and it is now complete.

l. 220. Please introduce RLC. I’m assuming retrogradely-labelled cells.

Reply: this has been done, please see pag. 6

l. 222. Which computer-based charting system? Please provide the software name or were in-house scripts

used?

Reply: Data from individual sections were then imported into the 3-dimensional (3D) reconstruction

software developed in house (Demelio et al., 2001), please see pags 6-7.

l. 280: Please clarify: Do the segments ‘dorsal, middle, ventral’ correspond to ‘dorsal bank, fundus of the

sulcus and ventral bank’? Or is this a segmentation only of the dorsal bank?

Reply: It is the segmentation of the dorsal bank of the IPS only, please see text at pag. 10.

l. 311: “ [...] normalizing the sum of the COMMIT weights of each ROI by the sum of COMMIT weights of all

ROIs of this sliding window”. Does this only refer to dorsal/middle vs ventral segment? Or all other cortical

ROIs? The entire paragraph is difficult to parse, please consider rephrasing in simpler words.

Reply: We have used the merged dorsal and middles sectors of our three-fold subdivision of the db-IPS for

the sliding window analysis. The connectivity distributions were computed between each window and all

cortical ROIs. We thank the reviewer for his comment, the paragraph has been rephrased for clarity (please

see text pag. 10).

Figure 2: were the red dots annotated manually or using the ‘computer-based charting system’ (see

above)? Some details on the definition of RCLs seems to be missing in the methods section.

Reply: The dots were annotated as follows: “In each examined section the outer and inner cortical borders

and the location of each labelled neuron were plotted with the aid of inductive displacement transducers

mounted on the X and Y axes of the microscope stage. The transducer signals were digitized and stored by

using software developed in our laboratory that allows the visualization of section outlines, of grey-white

matter borders, and of labelled cells. Data from individual sections were then imported into the 3-

dimensional (3D) reconstruction software developed in house (Demelio et al., 2001) to create volumetric

reconstructions of the hemispheres from individual histological sections containing connectional and/or

architectonic data and providing realistic visualizations of the labeling distribution.”, please see new etxt at

pag. 6-7

Figure 3: pre-CD is not labeled in the figure but referred to several times in the main text. Please consider

adding a label

Reply: the acronym pre-CD has been added

l. 441: Figure 7, should be Figure 6, I think.

Reply: This is correct, thank you! Correction has been made, please see pag. 143

l. 457: Fig. 8 should be Figure 7, I think etc, Figure numbers flipped several times, please update.

Reply: This is correct, thank you! Corrections have been made

-l. 518. It is noted that only dorsal and middle sectors are used, but further above it says that dorsal and

middle segment were merged (l. 304). This seems inconsistent, please clarify

Reply: Yes, correct, to take into account of this comment we have added the following sentence (please see

pag 17): “This choice was dictated by the histological verification of tracer injections, that did not involve

the deepest part of the dorsal bank, as well as by the fact that the latter can hardly be parcellated into three

dorso-ventral sections in its most anterior part, given the limited extent of the cortex in this dimension”. A

sentence was also added in the caption of fig. 11, to inform the reader.

l. 506. ‘the correlation between the distribution of RLC obtained after each injection sites and the

distribution of diffusion-based connectivity [etc...]’. I found this sentence (and also the next) hard to

understand and given that this is a key metric it should be rephrased so it is clear which numbers were

correlated. It only became clear to me when looking at Figure 9

Reply: We thank the reviewer for his comment. We have rephrased the paragraph for clarity, please see

pag. 17.

Is it not somehow an artifact that the correlation is maximal at the injection site? Please describe the

rational why this is an interesting finding and why this is not expected a priori.

Reply: The connectivity distributions of the db-IPS were obtained by two independent methods, on different

animals. This experiment tests the sensitivity of the DW-MRI connectivity in the fine parcellation of the db-IPS. Despite known limitations of DW-MRI connectivity analysis, such as the presence of false-positive

connections, Figure 8A shows it can indeed identify changes in the connectivity distribution that are

correlated with changes observed using RLC analysis. We have clarified this in the manuscript at pag. 17.

Figure 8B: Please describe more clearly what is shown. The figure displays a 3D rendering of the dorsal bank

of the db-IPS, but on a first glance I mistook it for an inflated brain surface. I’m not convinced this sub-figure is adding much compared to Figure 8A the way it is currently described in the main body.

Reply: Please notice that we have used this figure to “project” in a rigorous fashion the correlations values

in the anatomical MRI space of the dorsal bank of the IPS, as now specified in the text, please see pag. 17.

Figure 9: A colour legend is missing. I would also suggest touse a different colour mapping as it’s not clear

why both pPEip and aMIP are shown in blue.

Reply: Yes, thank you for noticing this problem. We have changed the color code in this figure, as well as in

those related to it (Figs 6,7,8).

Figure 12. Could a similar summary figure be constructed for the tract-tracing data? This would visualize

the finding that the two modalities produce results that are in part overlapping, but also diverge.

Reply: Unfortunately, this is not possible. Indeed, our tract tracing study is based on retrograde tracer

injections in the db-IPS and analysis of the labeled cells observed in the connected areas. In order to make a

figure similar to Figure 12, it would be necessary to inject retrograde (or anterograde) tracers within the

areas indicated in the Figure and show the retrograde (and/or anterograde) labeling within the db-IPS. 4

REVIEWER #2

ADVANCES THE FIELD This combined histological and MRI diffusion based tractography approach

constitutes a valuable resource for the community, since it provides a gold standard cross-validation of the

relationship between proportion of labelled cells projecting to PEip and MIP, and measures of diffusion

tractography connectivity. Furthermore, the authors demonstrate a significant correlation between the

proportion of cells projecting to MIP and/or PEip, and the diffusion-based connectivity. estimates of the

corresponding streamlines.

COMMENTS

It is not clear to me how authors identified the cytoarchitectonic areas described in the results section as

containing retrogradely labelled cells. A histological cell body staining? Neu-N immunohistochemistry? I did

not find this information in the section dedicated to histological procedures and data analysis.

Furthermore, although PEip and MIP can be distinguished based on their corticospinal connectivity, the

same does not apply to their rostrocaudal subdivisions. Thus, figures depicting their cytoarchitecture to

confirm identification would be helpful. The same applies to areas with. retrogradely labelled neurons. How

do the connectivity patterns depicted in Figure 12 correlate with cytoarchitectonically identified areas? The

authors should delineate their cytoarchitectonically identified areas - or provide an additional panel with a

schematic representation. Do their findings reconcile the differences between the map of Pandya and

Seltzer (1982) and that of Niu et al. (2020)?

Reply: We have better explained the reference maps for the cortical regions in which labeling was observed

(please see pag.7). Furthermore, please note that in the present study we have not proposed a subdivision

of MIP and PEip into architectonic distinct sectors, but we simply described connectional differences

between the rostral and the caudal part in both these areas. Furthermore, based on tractography we have

observed dorsoventral connectional gradients. In this respect, we have mentioned in the Discussion that

dorsoventral chemiiarchitectonic differences have been observed by Niu et al (2020).

MINOR POINTS

The legend to figure 1 is incomplete. This also makes interpretation of Figs 2 and 3 a bit difficult, as they

refer to the abbreviation list of Fig. 1.

Figure 12 would benefit from the labelling of areas within the region of interest

Reply: Thank you for the suggestion. Problem is that this image shows in, the MRI space of the dorsal bank

of the IPS, a gradient-like organization of the connectivity visualized by the tractography analysis. This

prevented to use the criterion of connectivity as marker of areal boundaries. We are afraid that drawing

lines between aPEip, pPEip, aMIP, pMIP on this native MRI images could introduce an element of

arbitrariness, since this information is, per se, not directly stemming from the data. In fact, we found a

smooth transition in the strength of connectivity in both the A-P and D-V dimensions of the dorsal bank,

which fits the physiological studies referred to in both the Introduction and Discussion. Therefore, although

we would have liked to adhere to the reviewer’s very reasonable request, we have elected to maintain the

figure as it is, since in this case we believe that less is better and more adherent to the results. We are sure

the reviewer will understand the choice to be very conservative.

There are some typos:

- Line 154, “Oxygen/ Isoflurane” should be “Oxygen/Isoflurane"

- Line 393, “very few. RLC were observed” should be “very few RLC were observed"

- Line 1064, “db_IPS” should be “db-IPS

Reply: Thank you, all typos have been corrected.
